# Templated Synthesis of Magnetic Nanoparticles through the Self-Assembly of Polymers and Surfactants

**DOI:** 10.3390/nano4030628

**Published:** 2014-08-04

**Authors:** Vo Thu An Nguyen, Mario Gauthier, Olivier Sandre

**Affiliations:** 1 University of Bordeaux, Laboratoire de Chimie des Polymères Organiques (LCPO), UMR 5629, F-33600 Pessac, France; E-Mail: annguyen@enscbp.fr; 2 National Center for Scientific Research (CNRS), LCPO, UMR 5629, F-33600 Pessac, France; 3 Department of Chemistry, University of Waterloo, Waterloo, ON N2L 3G1, Canada; E-Mail: gauthier@uwaterloo.ca

**Keywords:** superparamagnetic nanoparticles (NPs), templated synthesis, size and shape control, *in situ* synthesis

## Abstract

The synthesis of superparamagnetic nanoparticles (NPs) for various technological applications continues to be an interesting research topic. The successful application of superparamagnetic NPs to each specific area typically depends on the achievement of high magnetization for the nanocrystals obtained, which is determined by their average size and size distribution. The size dispersity of magnetic NPs (MNPs) is markedly improved when, during the synthesis, the nucleation and growth steps of the reaction are well-separated. Tuning the nucleation process with the assistance of a hosting medium that encapsulates the precursors (such as self-assembled micelles), dispersing them in discrete compartments, improves control over particle formation. These inorganic-organic hybrids inherit properties from both the organic and the inorganic materials, while the organic component can also bring a specific functionality to the particles or prevent their aggregation in water. The general concept of interest in this review is that the shape and size of the synthesized MNPs can be controlled to some extent by the geometry and the size of the organic templates used, which thus can be considered as molds at the nanometer scale, for both porous continuous matrices and suspensions.

## 1. Introduction

The synthesis of superparamagnetic iron oxide nanoparticles (SPIONs) and other transition metal (e.g., cobalt) ferrite nanoparticles (NPs) targeting various technological applications such as magnetic storage media, magnetic actuators or biosensors is a topic of ongoing interest for many researchers. In the biological and medical fields, magnetic NPs (MNPs) with a large specific surface area provide innovative potential in targeted drug delivery, in magnetic resonance imaging (MRI) as both positive and negative contrast agents, and in the magnetic hyperthermia treatment of tumors. The successful application of SPIONs in each specific area depends on the attainment of high magnetization values for the nanocrystals obtained, which is necessarily determined by their average size, size distribution, and crystallinity (absence of defects in the magnetic order). Additionally, to facilitate their biomedical applications, the nontoxicity, biocompatibility and stability of the coated magnetic particles in biological media have also been under the topics of numerous studies.

MNPs can be synthesized by numerous chemical techniques such as the alkaline coprecipitation of aqueous salts, hydrothermal reactions, microemulsion and sol-gel syntheses, sonochemical reactions, the hydrolysis and thermolysis of precursors, flow injection and electrospray syntheses, *etc.* Irrespectively of the method used, the formation of superparamagnetic NPs always involves nucleation and growth steps. Their separation is extremely difficult in the aqueous coprecipitation route, involving complicated pathways through oxo-hydroxide phases and eventually to magnetic iron oxide nanocrystals [1]. The size dispersity of the NPs prepared is markedly improved when these two steps are well-separated, which is unfortunately hard to achieve without relying upon kinetic parameters (for example, the temperature or mixing conditions) [2]. These barriers have been investigated and might be overcome by tuning the nucleation process with the assistance of a hosting medium, that encapsulates the precursors and disperses them in discrete compartments (such as small molecule or polymeric micelles), thereby providing control over particle formation. These inorganic-organic hybrids are particularly interesting since they inherit the properties of both the polymer and the inorganic material. In addition, coating by polymer chains can efficiently hinder aggregation of the particles [3]. The focus of this review is on methods providing some degree of control over the shape and the size of the synthesized NPs through variations in the geometry and size of the organic templates. Two classes of molds at the nanometer scale can be considered, consisting of either pores contained within a continuous matrix, or of individual objects suspended in a reaction medium. The organic component can also bring other advantages in addition to colloidal stability in aqueous media, such as the presence of specific functionalities.

## 2. *In Situ* Synthesis in Non-Polymeric Templates

Complexing groups such as the carboxylate, phosphate, sulfonate, and sulfate functionalities are known to bind strongly to the surface of magnetite. Furthermore, surfactants containing such head-groups can be tailored for stabilization in oil or hydrocarbon carrier fluids, as well as with charged ligands (citrate, oxalate, *etc.*) for aqueous media. The same type of moieties can used to complex the divalent or trivalent iron precursors selected for the reactions leading to the MNPs, for example by the coprecipitation of Fe^2+^ and Fe^3+^ salts in water or in a polyol medium, or the oxidation, reduction, or thermal decomposition of a Fe(II/III)-ligand complexes in a high boiling point solvent.

### 2.1. Carboxylates

#### Oleic Acid Coating in Combination with Reduction

**Figure 1:**
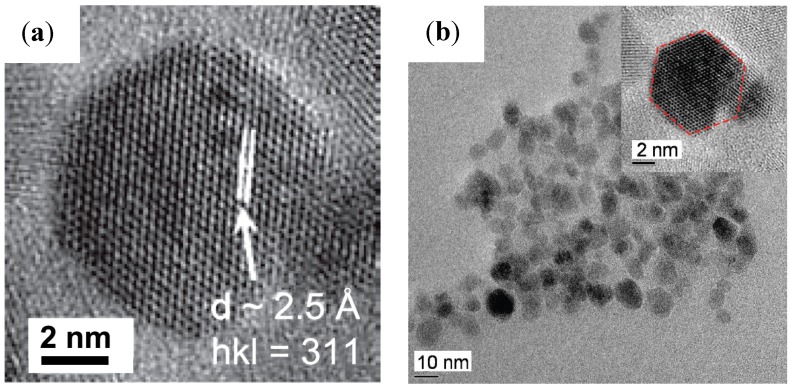
Transmission electron microscopy (TEM) images clearly showing: (**a**) lattice fringes; and (**b**) facetted shapes for nanoparticles (NPs) coated with oleic acid, using 10 equivalents of reducing agent (NaBH_4_) at room temperature. Reprinted with permission from [4]. Copyright 2011 WILEY-VCH Verlag GmbH and Co. KGaA, Weinheim.

The templating ability of organic compounds bearing carboxylic functional groups, for example oleic acid and l-glutamic acid, was investigated in the aqueous phase reduction/hydrolysis of iron (III) acetylacetonate (acac) using NaBH_4_ [4]. The ratio of reducing agent to iron precursor required to produce the largest diameter bare NPs (8.08 ± 0.93 nm by transmission electron microscopy (TEM) and 8.95 nm by X-ray diffraction (XRD) was first determined and then applied to the *in situ* coating method. Both the uncoated and the coated NPs displayed clear lattice fringes in high resolution transmission electron microscopy (HRTEM) images (Figure 1a) with spacings of 2.9 Å and 2.5 Å, consistent with a cubic spinel iron oxide structure and confirming the highly crystalline character of the NPs. It is noteworthy that some of the NPs coated with hydrophobic oleic acid had a facetted particle shape on the HRTEM images (insert on Figure 1b) rather than the spherical morphologies observed in most other cases, indicating that monolayer formation by the hydrophobic tail of oleic acid molecules brings a different templating effect as compared to several hydrophilic carboxylic compounds tested in the literature [5], most often leading to a mere reduction in the final size of the MNPs due to capping of the crystallites.

### 2.2. Sulfonates and Sulfates

#### 2.2.1. Sodium Dodecyl Sulfate (SDS) Microemulsions

The use of non-polymeric templates in MNP synthesis was actually investigated earlier by the group of Liu [6], who performed CoFe_2_O_4_ NPs synthesis by a microemulsion process with SDS as a surfactant. The size of the CoFe_2_O_4_ NPs was successfully controlled in a range from less than 4 nm to approximately 10 nm, by varying the concentration of metallic precursors and the methylamine base added [6]. Interestingly, the influences of NP size and temperature on the magnetic properties were systematically studied. For example, a decline in the remnant magnetization of 8.5 nm CoFe_2_O_4_ NPs with increasing temperature and its disappearance leading to the demagnetization of the NPs at 290 K were noted. Using field cooled (FC)/zero field cooled (ZFC) superconducting quantum interference device (SQUID) magnetometry, the magnetic susceptibility of NPs of different sizes was shown to exhibit the usual temperature dependence: initially increasing as the temperature was lowered, it reached a maximum at the point referred to as the blocking temperature (*T*_B_), and eventually decreased. A correlation between higher *T*_B_ and increasing NPs diameters was convincingly pointed out. The superparamagnetism of the NPs above their *T*_B_ was confirmed by neutron diffraction measurements, while the increases in the *T*_B_, coercivity (*H*_c_) and saturation magnetization (*M*_s_) with the size of the particles were in agreement with the Stoner-Wohlfarth theory on single domain particles. Such a study could not have been completed without the exceptionally good control achieved over the size and size dispersity by the microemulsion templating process.

#### 2.2.2. Sodium Dodecylbenzenesulfonate (NaDBS) Microemulsions

The same technique to control the size of MNPs by varying the relative concentrations of iron salts, surfactant, and solvent in a water-in-oil microemulsion system was demonstrated by Lee *et al.* [7]. This effective approach using NaDBS microemulsions as nanoreactors and hydrazine as precipitating agent allowed the size of the NPs to be tuned in the range of 2–10 nm with narrow size distributions and high levels of crystallinity. The lattice fringes appearing in HRTEM imaging again hinted at the highly crystalline structure of the MNPs obtained. The matching XRD patterns obtained for the MNPS and for a magnetite standard further confirmed the structure of the Fe_3_O_4_ phase, while the peak widths calculated from the Debye-Scherrer equation were consistent with the observed TEM sizes. The magnetization *versus* temperature curves followed a pattern similar to the one previously described, in which the *T*_B_ and *M*_s_ for larger particles had higher values. These results were in good agreement with the Stoner-Wohlfarth theory in terms of the increase in activation energy for larger particles, although determining the origin of the smaller *H*_c_ values observed as compared to related studies would require further investigation. More importantly, the procedure was taken a step further by producing several mixed metal ferrites in a size range of 4–8 nm, with uniform particle size distributions and an inverse spinel structure. 

## 3. *In Situ* Synthesis with Polymers in Solution or in the Bulk State

The preparation of aqueous magnetic fluids was been achieved more recently using polymers as steric stabilizers in solution and polymer templates in the bulk state. These studies will be presented according to the different types of polymers used for that purpose.

### 3.1. Dextran (DEX) and Polysaccharide Derivatives

The term DEX refers to a class of branched polysaccharides composed from a majority of α-1,6- and a minority of α-1,3- or α-1,4-glycosidic linked glucose units, which are non-toxic, biocompatible and biodegradable hydrophilic polymers extensively used in biomedical applications and in NP preparation procedures. The general role of DEX in enhancing the biocompatibility of metal oxides, using green coprecipitation and gel template strategies, was previously discussed in a review by Patrinoiu *et al.* [8]. For the purposes of this discussion, we are primarily interested in the efficiency of DEX in templating and controlling the shape and size of MNPs.

#### 3.1.1. Influence of DEX End-Group Reduction

In an attempt to optimize the nucleation of ultra-small superparamagnetic iron oxide (USPIO) particles in the presence of DEX, the effects of chemical functionalities and the molecular weight (MW) on the formation, stability, size, and magnetic properties of the hybrid particles obtained were investigated by Paul *et al.* [9]. Reduced DEX, generated by sodium borohydride treatment of native DEXs of different MW, has many hydroxyl groups existing in the native structure but only one additional hydroxyl group converted from the aldehyde (hemi-acetal) terminus. Despite this minor change in structure, reduced DEX was reported to successfully produce USPIOs with a volume-average diameter of less than 30 nm, while the size of native DEX-coated particles synthesized in the same conditions was larger than 700 nm in most cases and even reached 2.7 µm for 1 kDa DEX. Samples prepared with low MW reduced DEX (1 kDa and 5 kDa) possessed a magnetic susceptibility 5–25 times stronger than their native counterparts. However, the influences of the reduced terminal unit on the size and the magnetic susceptibility of the USPIOs were diminished for DEXs of higher MW.

The minor change at the terminal sugar unit of 10 kDa MW DEX resulting from the reduction reaction also led to more efficient utilization of the templating material, as more stable autoclaved USPIOs with much smaller diameters (23 nm *versus* 587 nm) were formed, with a higher percentage of metal-bound DEX (0.96 g DEX/g Fe *versus* 0.59 g DEX/g Fe).

The size effect of reduced 10 kDa DEX was emphasized through similar experiments performed with 2.5–50 wt% addition of the simple sugar glucose *versus* the amount of DEX used. The samples derived from reduced DEX with added glucose had radii similar to those prepared without sugar added, thus convincingly demonstrating binding enhancement arising from the additional OH groups in the linear polyol termini as compared to the aldehyde functionality present in the monosaccharide. It is worth mentioning that the size of the USPIO particles increased with the MW of DEX (20 nm for 1 kDa *versus* 41 nm for 70 kDa DEX). However, stable colloids could only be created using reduced polysaccharides having a MW larger than 10 kDa as compared to 70 kDa for native DEX. This shows the dominant role of the MW of the chains in the growth of larger colloids from an initially unstable magnetic sol.

#### 3.1.2. DEX Sulfate

Beyond the encapsulation enhancement brought by reduced DEX as compared to the native material, further modifications of this type of material may provide additional options leading to improved physicochemical properties. In addition to being considered a ligand for macrophage scavenger receptors, DEX sulfate was also proposed as an advanced coating material as compared to reduced DEX: a mixture of only 5 wt% DEX sulfate with reduced DEX yielded particles with a hydrodynamic diameter of over 30 nm and insignificantly larger core sizes (7.9 ± 2.0 nm *versus* 7.0 ± 1.9 nm for reduced DEX) [10]. Experimental parameters including the reaction time and the pH were varied to create DEX sulfate-coated NPs with good magnetic resonance properties. The delayed addition of Fe^2+^ was mentioned as a technique to avoid overgrowth and crosslinking of the particles caused by interactions between excess Fe^3+^ and the polysaccharide coating. While an increase in the polysaccharide/Fe^3+^ ratio decreased the core size and thus the relaxivity of the particles, failure of particle formation was also reported at very low polysaccharide concentrations. 

#### 3.1.3. DEX-*b*-poly(methacrylic acid)(Fe_3_O_4_)-*b*-Poly(*N*-Isopropylacrylamide) with Thiol End Groups

The synthesis of quadruple-responsive nanocomposites was achieved in solutions of the copolymer DEX-*b*-poly(methacrylic acid)-*b*-poly(*N*-isopropylacrylamide) (DEX-poly(methacrylic acid)-PNiPAAm) where complexation was achieved by the poly(methacrylic acid) (PMAA) block, the PNiPAAm units provided thermo-sensitivity, the iron oxide NPs (IONPs) offered the magnetic response, and nucleated gold nanorods (AuNR) triggered near infrared radiation (NIR) response [11]. The diameter of the SPIONs before the AuNR encapsulation was 10 ± 3.5 nm (based on TEM analysis) with a narrow size distribution, while the aqueous hydrodynamic radius (*R*_h_) measured by dynamic light scattering (DLS) was 66–69 nm, suggesting that a thick copolymer shell covered the SPIONs. The dependence of the lower critical solution temperature (LCST) on pH (29 °C at pH 4.5 *versus* 35 °C at pH 12) was explained by changes in the hydrophobic/hydrophilic balance caused by protonation of the PMMA blocks. Vibrating sample magnetometer (VSM) measurements at room temperature revealed superparamagnetic properties with neither remanence nor *H*_c_. At room temperature, SPIONs 10 ± 3.5 nm in size had limited stability in a 300 mT magnetic field, displaying slow aggregation under these conditions. The magnetic interactions were strongly enhanced above the LCST, however, (e.g., at 50 °C), due to the phase transition of the hybrids that induced aggregation via hydrophobic as well as magnetic dipolar interactions. Owing to the presence of the PNiPAAm blocks the phase transition was reversible, providing water solubility to the recovered SPIONs when the temperature was brought back below the LCST, due to the hydrophilic DEX units.

#### 3.1.4. Alginate (Alg) Beads

**Figure 2:**
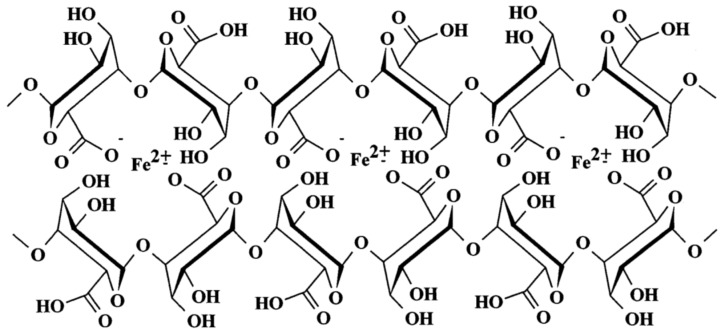
Representation of two 1,4-linked α-l-guluronic acid (G)-blocks forming an “egg box” structure with ferrous ions. Reprinted with permission from [9]. Copyright 2000 Elsevier Science B.V.

As a naturally occurring, biocompatible and inexpensive polysaccharide widely used in encapsulation processes, alginate is constituted of 1,4-linked α-l-guluronic acid (G) and 1,4-linked β-d-mannuronic acid (M) units (Figure 2), either segregated into homopolymer blocks or combined in copolymer structures. Commercially available alginates extracted from the cell wall of either brown algae or bacteria have different M/G compositions, MW, sequences, and physicochemical properties which strongly affect their efficiency in encapsulation applications [12].

The loading capacity of alginates with different M/G ratios was investigated in the *in situ* alkaline oxidation of ferrous ions to produce maghemite MNPs [9]. More precisely, this synthesis relied upon the alkaline coprecipitation of Fe^2+^ and Fe^3+^, but with the particularity of the *in situ* production of Fe^3+^ ions by the oxidation of Fe^2+^ in air. After a first oxidation cycle, a higher iron content (*ca*. 31%) was recorded in a sample for which the M/G ratio was 0.6, as compared to *ca*. 14% for the alginate with M/G = 1.6. This result highlights the superior binding ability of guluronic *versus* mannuronic acid units resulting from the “egg box” conformation of the guluronic acid segments that trap the Fe^2+^ ions more efficiently. Larger *M_s_* values were recorded for the M/G = 0.6 samples, obviously due to their higher total iron content. As the total iron content in two samples reached the same maximum value after three oxidation cycles (approximately 47.09% *versus* 48.29%), these two samples displayed similar magnetization curves. The superparamagnetic character of these fully oxidized samples at room temperature was confirmed by their large values of *M*_s_, zero hysteresis, and from the paramagnetic Mössbauer spectra obtained. Although no further data were provided about the size and the morphology of the maghemite NPs obtained, the hosting ability of alginate compounds was clearly demonstrated.

#### 3.1.5. Precipitation in Aqueous Alginate Networks

Recently, questions related to the size and morphology of MNPs encapsulated in alginate networks were convincingly answered by the team of Srivastava *et al.* [12] who took the above approach one step further with the use of sodium alginate from *Laminaria hyperborea* (MW = 60,000–80,000 g∙mol^−1^, M/G = 32/68) to synthesize Fe_3_O_4_/Alg nanocomposites with a core-shell structure in a controlled low-temperature oxidation/precipitation process at pH 4, 10 and 14. The Fe loading, the size and, more interestingly, the magnetic properties of the core-shell composites were efficiently controlled through the final pH used in the precipitation process. It was indeed reported that higher Fe loading could be achieved at low pH (4–5) than at high pH (14). The Fe_3_O_4_ content of the alginate composites synthesized at pH 4–5 was confirmed by XRD analysis. Calculation of the crystallite size from the XRD patterns gave a mean value of *ca*. 14 nm, while the values determined using the Scherrer equation and in TEM micrographs were *ca*. 10 nm and 10.5 nm, respectively. As compared to bare Fe_3_O_4_ NPs, the alginate composites exhibited better dispersibility due to the alginate shell, essentially reducing the interactions between the MNPs. While neither *H*_c_ nor remnant magnetization were detected in the *M-H* curves for the magnetite alginate composites, confirming their superparamagnetic behavior, the saturated magnetization values exhibited by the hybrids were much lower (below 6 emu/g) than for pure Fe_3_O_4_ NPs prepared by the same process (above 31 emu/g). This was assigned to differences in morphology among these two sample series: the Fe_3_O_4_ synthesized at pH 4 was better dispersed than the sample produced at pH 10, which was agglomerated to some extent. The *M*_s_ values for the composites were larger for the NPs synthesized at a higher pH, which is obviously in agreement with the Fe loading efficiency reported above.

### 3.2. Synthetic Linear Homopolymers

To maximize the *in situ* templating effect as well as to enhance the stability of the ferrofluids obtained, numerous attempts have been made in terms of: (i) changing the chemical nature of the template material; and (ii) varying the method employed to produce the NPs. The first approach is more convenient experimentally for synthetic linear homopolymer templates than for natural polysaccharide derivatives, due to the fact that the former materials can possess controlled chemical structures, MWs, and MW distributions, which facilitates magnetic property improvement and particle surface tailoring in terms of enhanced colloidal stability and biocompatibility. 

#### 3.2.1. Thermal Decomposition of Poly(Vinyl Alcohol)-Fe(OH)_3_

One of the first synthetic homopolymers investigated as template for the preparation of MNPs was poly(vinyl alcohol) (PVA), containing a large number of hydroxyl functional groups available to interact with Fe^3+^ ions and leading to water-soluble complexes. In their pioneering work, Yokoi and Kantoh [13] successfully combined different amounts of PVA to Fe(OH)_3_ and obtained Fe_3_O_4_ precipitates by thermal decomposition. Although agglomerates of sintered magnetite particles were obtained instead of uniform products, the diameter of the Fe_3_O_4_ crystals in the composites evaluated from the line width of the (311) diffraction peak linearly increased with the heating temperature, indicating that the growth of Fe_3_O_4_ crystals at higher temperatures, under the reducing effect of PVA, converted one third of the Fe^3+^ into Fe^2+^, as required by the stoichiometry of the coprecipitation reaction.

#### 3.2.2. Fe^2+/3+^ Coprecipitation in PVA Membranes

In continuation of the earlier work using PVA solutions as *in situ* templates, Sairam *et al.* [14] performed the alkaline coprecipitation of various amounts of Fe^2+^ and Fe^3+^ previously impregnated in PVA membranes. The free-standing composite membranes prepared were expected to be useful as pervaporation separation systems to dehydrate organic solvents. The encapsulation of IONPs in PVA increased the mechanical strength of the membranes, but also enhanced their water affinity as shown by the improvement in solvent drying performance with increasing iron oxide content.

#### 3.2.3. Conversion of Fe^3+^ in PVA into Hematite in Supercritical H_2_O

Another study dealing with antiferromagnetic iron oxide (α-Fe_2_O_3_ or hematite)-PVA composites was conducted by Xu and Teja [15] using continuous hydrothermal processing (CHP) in supercritical H_2_O, a process allegedly providing size and morphology control for metal oxide NPs. Although the influence of the experimental variables on the particle characteristics was not convincingly determined, a higher ferric nitrate concentration, longer residence time, or elevated temperature did result in increased average particle radii and crystallinity, as confirmed by narrowing of the peaks in XRD patterns. A larger average particle size (27.4 nm *versus* 15.6 nm) and morphological transformations from spherical to rhombic (elongated) shapes were also reported as a consequence of a higher precursor concentration (0.06 M *versus* 0.03 M). More importantly, the particle size and size distribution (standard deviation) both decreased as the PVA concentration increased, as demonstrated in three experiments with a reaction time of 2.6 s (controlled by the residence time in the high pressure conducts). As the molar ratio of vinyl alcohol units to Fe^3+^ in the starting mixture was increased from 0:1 to 1:1 and 2:1, the average particle size of the samples decreased from 15.6 nm to 10.1 nm and 7.2 nm, respectively. This led the authors to conclude that a minimum amount of PVA was needed to coat all the particles, resulting in a polymeric layer with a thickness of approximately 10 nm that hindered further growth of the particles.

#### 3.2.4. Fe(acac)_3_ Decomposition/Diol Reduction in Octyl Ether (OE) with Polyvinylpyrrolidone (PVP)

It is widely accepted that the main limitation of the thermal decomposition of Fe(CO)_5_ to prepare monodispersed Fe_3_O_4_NPs is the high toxicity of the carbonyl precursor. In an attempt to solve this issue, Fe^3+^ acac was used in an one-pot polyol reduction process in OE and in the presence of a water-soluble polymer, PVP, playing the role of polymeric stabilizer [16]. The PVP-coated, monodispersed Fe_3_O_4_ NPs obtained had diameters <5 nm and displayed well-defined superparamagnetic behavior at room temperature with zero *H*_c_.

#### 3.2.5. Controlled Precipitation in the Presence of Poly(4-vinylpyridine) (P4VP)

The role of polymers containing nitrogen-based functional groups in the synthesis of MNPs was furthered investigated in the *in situ* controlled precipitation of Fe^3+^ ions in Fe^3+^-P4VP coordination complexes prepared beforehand in water/acetone mixtures and dried at 60 °C [17]. In this approach, the iron-P4VP solid matrix played the role of a growth-restricted medium, where ion diffusion was efficiently slowed down and the growing iron NPs were effectively “trapped” due to Fe–N interactions with the pyridine groups. More importantly, the hydrophilicity of protonated P4VP before precipitation, that facilitated N-Fe coordination, was described as an important factor controlling particle growth. At the onset of precipitation, the protonated pyridine groups became hydrophobic, inducing the collapse of the homogeneous iron-polymer gel and the creation of a non-uniform microstructure consisting of hydrophilic and hydrophobic regions. The subsequent growth process was restricted by the amount of Fe ions available only in the hydrophilic regions, which led to small particles. The hybrid polymer nanocomposites obtained after washing and heating to 150 °C yielded rod-like NPs in TEM images. These nanorods, with lengths ranging from 15 nm to 75 nm and antiferromagnetic properties affected by the Cl/Fe atomic ratio, consisted of the antiferromagnetic chlorinated iron oxy-hydroxide “akaganeite” (Fe_8_O_8_OH_8_Cl_1.35_, also noted β-FeOOH). It was speculated that incomplete filling of the Cl^−^ sites affected the perfect compensation of antiferromagnetic sublattices, resulting in the appearance of a magnetic moment. Perfect sublattice alignment is indeed only achieved in a Cl^−^-saturated crystal lattice (*i*.*e*., in a highly acidic P4VP environment). In that particular case, the templating effect of the polymer on the NPs can be partially ascribed to the chlorine counterions of this weak polyelectrolyte.

#### 3.2.6. Poly(ethylene oxide) PEO in the “One-Pot” Synthesis of Pegylated Oxide NPs

Being known for its excellent solvating characteristics, good complexing ability for several transition metal cations, exceptional resistance to oxidation, reduction, and decomposition (by acids and bases at moderately high temperatures, hydrogen peroxide, and sodium borohydride), and the possibility of end-functionalization, poly(ethylene glycol) (PEG) or PEO has naturally become a good candidate to orient the synthesis of magnetic particles. Its stealth characteristics, that is, the ability to avoid non-specific interactions with proteins and uptake by the reticulo-endothelial system, provides to NPs pegylated on their surface a simple route for further medical applications [18].

#### 3.2.7. PEO in Alkaline Hydrothermal Treatment

**Figure 3:**
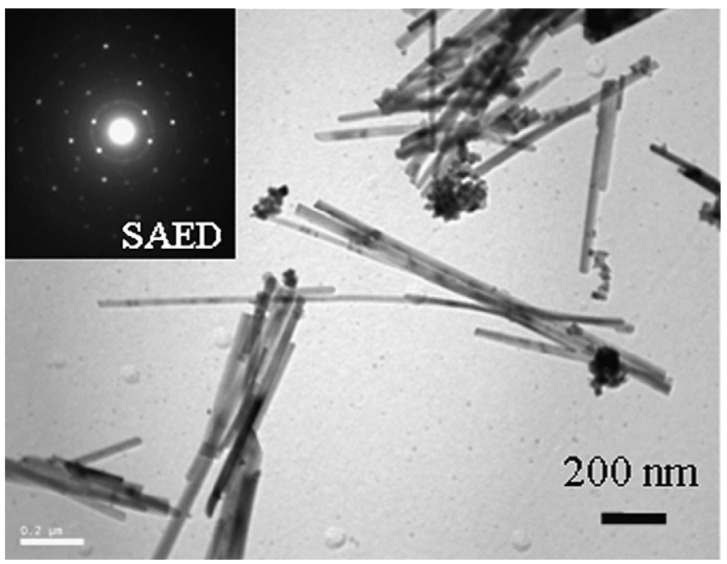
TEM image for magnetite nanowires synthesized with poly(ethylene glycol) (PEG)/H_2_O (1:2 by volume) at 150 °C for 24 h in an autoclave. The selected area electron diffraction (SAED) pattern is shown in the inset. Reprinted with permission from [19]. Copyright 2008 Elsevier B.V.

While conventional templating methods often result in the formation of polycrystalline nanowires or nanorods, monocrystalline 25 nm × 1.5 µm magnetite nanowires (Figure 3) were successfully produced in a PEG-assisted hydrothermal process [19]. There are numerous challenges in controlling the size, morphology, and crystallinity of Fe_3_O_4_ nanowires due to their complex inverse spinel structure. Full control over the iron oxide nanostructure phase through careful variations in the PEG and H_2_O concentrations and the reaction temperature was claimed (see Table 1 and Table 2). A maximum *M*_s_ value of ~91 emu/g, as obtained from vibrating magnetometer measurements, was achieved under the optimal conditions, confirming the crystalline perfection and extremely high purity of the sample (without γ-Fe_2_O_3_, having an *M*_s_ value in the bulk of 80 emu/g). The nanowire formation mechanism proposed involved PEG in a critical role as a stabilizer, mild reducing agent, and growth-orienting agent for the newborn Fe_3_O_4_ NPs into nanowires.

**Table 1:** Influence of the PEG:H_2_O volume ratio on the identified phases and crystallite size collected from X-ray diffraction (XRD) data. All samples are prepared at 150 °C for 24 h. Reprinted with permission from [19]. Copyright 2008 Elsevier B.V.

PEG:H_2_O (in volume)	Phases	Crystallite size (nm)
1:3	Fe_3_O_4_, α-Fe_2_O_3_	21.8
1:1	Fe_3_O_4_, α-Fe_2_O_3_, γ-Fe_2_O_3_	24.7
3:1	Fe_3_O_4_, α-Fe_2_O_3_, γ-Fe_2_O_3_	31.6
0:4	Fe_3_O_4_, α-Fe_2_O_3_, γ-Fe_2_O_3_	42.2
4:0	NaFeO_2_	64.5

**Table 2:** Influence of reaction temperature and time on the phases of nanostructured iron oxides and crystallite sizes. All samples are prepared using PEG:H_2_O = 1:2 in volume. Reprinted with permission from [19]. Copyright 2008 Elsevier B.V.

Conditions: temperature (°C)/time (h)	Phases	Crystallite size (nm)
100/24	Fe_3_O_4_ + NaFeS_2_(H_2_O)_2_	10.6
125/24	Fe_3_O_4_ + α-Fe_2_O_3_ + NaFeS_2_(H_2_O)_2_	13.8
150/24	Fe_3_O_4_	30.3
150/48	Fe_3_O_4_ + α-Fe_2_O_3_	24.1
150/72	Fe_3_O_4_ + α-Fe_2_O_3_ + γ-Fe_2_O_3_	28.4

#### 3.2.8. Poly(acrylic acid) (PAA) Chains for Fe_3_O_4_ Growth Inhibition

Up to this point, the majority of synthetic polymeric stabilizers discussed did not contain functional groups able not only to bind efficiently to the surface of Fe_3_O_4_ particles, but also to add strong enough repulsions (electrostatic and/or steric) between the MNPs and lead to an enhanced colloidal stability desirable for true “ferrofluid”. In a search for different homopolymers with suitable functional groups, Lin *et al.* [20] investigated PAA MW~2000 g∙mol^−1^ whose carboxylic acid groups can theoretically form strong ionic bonds with Fe_3_O_4_, and thus act as template for iron oxide nucleation. Furthermore, the presence of excess COOH groups at the surface of the coated particles should provide both electrostatic and electrosteric stabilization enhancing the stability of aqueous magnetite suspensions.

The negative zeta potential obtained from laser light scattering velocimetry measurements demonstrated that the polymer molecules could bind to the surface of Fe_3_O_4_ NPs (particularly at alkaline pH), providing both electrostatic and steric stabilization against particle aggregation. This repulsion (especially at high concentrations of stabilizing polymer) sterically hindered the growth of the particles, producing much smaller products with narrow size distribution, as compared to traditional methods.

Pseudo-aggregates of NPs, with a size of about 150–450 nm, were also observed several weeks after the ferrofluids were synthesized. The explanations suggested for this were hydrogen bonding between the COOH groups, or the entanglement of the PAA polymers when present at a high concentration in the synthesis. The fact that these large, broadly distributed aggregates were destroyed by sonication before light scattering measurements was confirmed by the appearance of 10–40 nm ferrofluid species with much narrower size dispersity. The *M*_s_ for the PAA-coated ferrofluid was about 35 emu*/*g, to compare with 50 emu*/*g for pure Fe_3_O_4_ NPs. The reduced value of *M*_s_ was explained by the presence in the particles of γ-Fe_2_O_3_ (which is ~20% less magnetized than pure Fe_3_O_4_) and by the significant reduction in *M*_s_ expected for particle sizes below 10 nm.

#### 3.2.9. FeCl_3_ Thermal Decomposition with PEG in Triethylene Glycol (Polyol)

**Figure 4:**
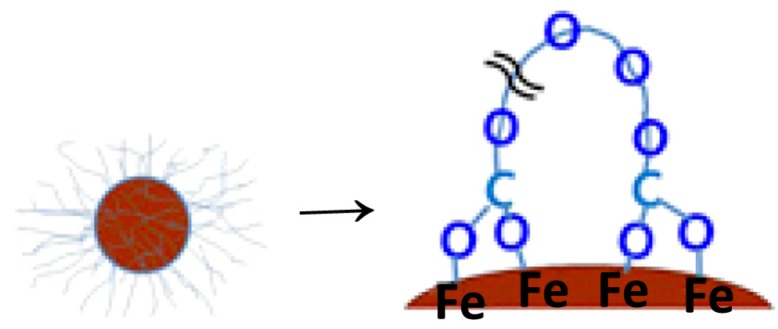
Suggested bond formation between COO^−^ and the Fe^3+^ ions at the ultra-small superparamagnetic iron oxide (USPIO) surface. Reprinted with permission from [21]. Copyright 2008 IOP Publishing Ltd.

In another study, PEG with carboxylic acid functionalities at both chain ends (PEG diacid, *M_n_* ~ 600) was employed in a one-pot synthesis in a polar organic solvent, triethylene glycol (triEG), to yield surface-modified ultra-small SPIONs (USPIOs) with an average particle diameter (from TEM) of 1.7 nm [21]. These particles were nearly monodispersed and were highly water-dispersible, yielding a hydrodynamic diameter of about 5.4 nm, significantly larger than the diameter determined by TEM analysis (1.7 nm) due to the thickness of the hydrated PEG coating. The composition of USPIOs in the PEG-modified USPIOs, estimated from thermogravimetric analysis (TGA), was 30.65% and was used to correct the net magnetization values to yield 23.5 emu/g and 6.6 emu/g at *T* = 5 K and 300 K, respectively. Additional data obtained from the *M*-*H* curve indicated that the USPIOs were ferromagnetic at 5 K and superparamagnetic at 300 K. Fourier transform infrared spectroscopy (FTIR) spectra for PEG and the PEG-modified USPIOs exhibited a C–H stretch at 2890 cm^−1^, and a C–O stretch at 1110 cm^−1^ in both cases, confirming the presence of PEG in the composites. More importantly, in the spectrum for the PEG-USPIO composites, the C=O stretch at 1745 cm^−1^ in PEG was not prominent and was shifted to 1645 cm^−1^ region, which suggests bonding between COO^−^ (rather than –O– of the polyether) and Fe ions on the surface of the particles, as shown on Figure 4. Relaxivity measurements and magnetic resonance images obtained highlighted the potential usefulness of these particles as both *T*_1_ and *T*_2_ MRI contrast agents, due to their ultra-small size, and the possibility of achieving target-specificity and a long blood half-life. 

#### 3.2.10. Coprecipitation in the Presence of Trithiol-Terminated Poly(methacrylic Acid) (PMAA-PTTM)

Another homopolymer containing multiple carboxylic acid groups is PMAA. A dual-functionality PMAA with trithiol chain ends (PMAA-PTTM) was used in a single-step aqueous coprecipitation procedure leading to narrowly distributed Fe_3_O_4_ nanocrystals [22]. Interestingly, an increase in the molar ratio of COOH to the Fe (II) and Fe (III) ions (from 0.7 to 3.4) clearly led to a decrease in size (from 6.1 nm to 4.5 nm) as well as narrowing of the size distribution of the particles (standard deviation decreasing from 1.1 nm to 0.4 nm), thus indicating the dominant role of the COOH groups *versus* trithiol termini of the polymer in controlling the nucleating process. The less influential role of the trithiol chain ends in the impregnation step offers a facile post-synthesis functionalization path to the introduction of more reactive groups with further conjugation capacity, without causing large changes in size, size distribution or water-solubility of the NPs. The coated Fe_3_O_4_ nanocrystals had a size of 4.5 nm, clearly exhibited superparamagnetic characteristics on the *M-H* isotherms measured at 300 K, and possessed calculated *M*_s_ of 48 emu/g Fe_3_O_4_, which is surprisingly high as compared with conventional magnetite particles of the same size (*M*_s_ < 15 emu/g). This low magnetization is usually attributed to several factors such as crystallinity defects including non-stoichiometry of the spinel structure, surface spin-canting, surface disorder caused by interactions with surface ligands, cation site distribution, *etc.* In a study by another team [23], PMAA was also used to produce coated agglomerates by an aqueous coprecipitation method, having magnetic core sizes around 8 nm by TEM analysis and hydrodynamic mean sizes ranging from 1.5 µm to 3 µm. The PMAA-encapsulated magnetite particles did not exhibit superparamagnetic behavior however, but rather a small remnant magnetization and *H*_c_, probably due to the aggregation of the magnetite NPs, although the individual 8 nm magnetite NPs displayed superparamagnetic properties. A low magnetization of 40 emu/g determined at 7 T, as compared to the magnetization values for bulk magnetic iron oxides (92 emu/g for Fe_3_O_4_, magnetite, and 80 emu/g for γ-Fe_2_O_3_, maghemite), was assigned to the particle surface spin disorder effects listed above [23].

#### 3.2.11. Poly(Methyl Glutarimide) (PMGI) Templated Iron NPs by Spin-Coating

While investigating a novel method to generate high-density and uniformly distributed carbon nanotube (CNT) mats, Lu *et al.* [24] employed spin-coating to prepare a film of PMGI loaded with various iron precursors (Fe^3+^ chloride, nitrate or acac, and the organometallic complex ferrocene) for the templated formation of IONPs, subsequently exploited as catalysts for CNT growth. Following the trend of using polymeric templates with specific characteristics, PMGI, a polymer widely used in the bilayer lift-off process, was expected to form complexes with Fe^3+^ ions by multidentate coordination.

The particle size, distribution, and overall yield in the spin-coating process were strongly influenced by the complexation tendency of the iron precursors used with the PMGI templates. The particles derived from strongly coordinated compounds like ferrocene and iron acac had a low density of iron oxide and broad size distributions, since the Fe^3+^ ions in these materials were unable to complex with the PMGI chains due to steric hindrance caused by the bulky counterions. Iron nitrate and iron chloride produced the highest and second highest density of IONPs, respectively, with small mean diameters, since both monodentate anions were less bulky and more loosely bound to iron.

### 3.3. Synthetic Linear Copolymers

Block copolymers exhibiting microphase separation into ordered morphologies such as lamellar, cylindrical and spherical microdomains, provide a convenient self-assembled template for the synthesis of nanocomposites. These microdomains represent nanoreactors within which a variety of NP clusters can be synthesized.

#### 3.3.1. Aqueous Coprecipitation with Double-Hydrophilic Block Copolymers (DHBCs)

To investigate the templating effects of DHBCs as well as tailored functional groups on the surfaces, Wan *et al.* [25] prepared stable aqueous ferrofluids using copolymers with poly(glycerol monoacrylate) (PGA) or poly(glycerol methacrylate) (PGMA) as one block, and either PAA, poly[(*N*,*N*-dimethylamino)ethyl methacrylate] (PDMAEMA) or poly(ethylene glycol) monomethyl (mPEG) ether as the other block.

When coated with PAA-*b*-PGA the surface of SPIONs carries negative charges, while the surface of particles obtained from PDMAEMA-*b*-PGMA, of which the PDMAEMA chains are protonated at neutral pH, possesses positive charges.

Multidentate coordination arising from the formation of five-membered rings between the 1,2-diol groups by the PGA or PGMA blocks and Fe^2+^ or Fe^3+^ cations at the surface of the magnetic particles was proposed to be more stable than the four-membered rings formed by the COOH groups in PAA. The disappearance of the COO^−^ adsorption band at 1592 cm^−1^ in the FTIR spectra of PAA-*b*-PGA-coated IONPs indicated that the carboxylic groups of PAA-*b*-PGA were not coordinated with the Fe^2+/3+^ cations. Additionally, magnetic fluids obtained with PGA or mPEG-*b*-PGA were stable over a wider pH range as compared to those prepared from PAA or mPEG-*b*-PAA. PGA was considered as a better stabilizer than PAA for the dispersions, such that the PGA block of the copolymer was coordinated to the surface of the magnetite NPs, while the PAA block was extended into water.

The same conclusion was drawn in the cases of PDMAEMA-*b*-PGMA or mPEG-*b*-PGA block copolymers, of which the PGMA or PGA blocks were chemisorbed onto the surface of the IONPs, thus playing decisive role in orienting particle synthesis while the PDMAEMA or mPEG blocks, with a swollen conformation in water, contributed to post-synthesis stability.

#### 3.3.2. Aqueous Coprecipitation with Triple-Hydrophilic Block Copolymers (THBCs)

**Figure 5:**
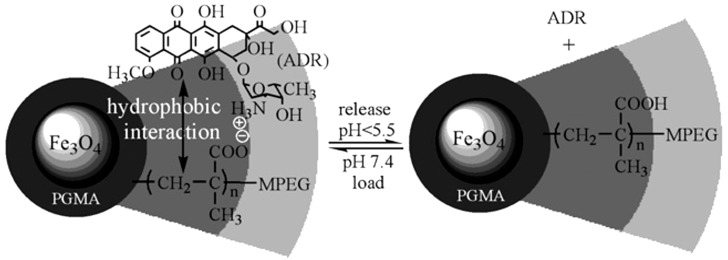
Schematic structure of mPEG-*b*-PMAA-*b*-PGMA-Fe_3_O_4_ NPs loaded with adriamycin at neutral pH, designed to release the anticancer drug in the acidic environment of a tumor. mPEG: poly(ethylene glycol) monomethyl; PMAA: poly(methacrylic acid); PGMA: poly(glycerol methacrylate); and ADR: antitumor drug adriamycin. Reprinted with permission from [26]. Copyright 2008 Royal Society of Chemistry.

Following this research trend, triblock copolymers constituted from the monomers listed above, but particularly mPEG-*b*-PMAA-*b*-PGMA, were used in the alkaline coprecipitation of Fe^2+^/Fe^3+^ [26]. PGMA influenced the nucleation step by tightly binding to the magnetite cores, while PMMA created an intermediate layer with a double function of bearing the mPEG corona and encapsulating the antibiotic and antitumor drug adriamycin (ADR), also called doxorubicin. The drug encapsulation was driven by both electrostatic interaction at pH 7.4 between anionic COO^−^ of PMMA and the protonated glycosidic amine of ADR (p*K*_a_ = 8.2) and hydrophobic interaction between the PMAA main chain and the hydrophobic anthracycline ring of ADR (Figure 5). The size of the Fe_3_O_4_ core was estimated to be about 7–8 nm from TEM micrographs, whereas a narrow size distribution and an average *R*_h_ of 23 nm were determined for the coated particles by DLS analysis. Superparamagnetic behavior at 300 K without magnetic hysteresis was observed for the MNPs obtained.

#### 3.3.3. Fe^2+^/Fe^3+^ Coprecipitation with Silicon-Containing Random Copolymers

The effectiveness of an amphiphilic random block copolymer of 3-(trimethoxysilyl)propyl methacrylate and ethylene glycol methyl ether methacrylate, denoted as poly(TMSMA-*r*-PEGMA), as stabilizer for MNPs was assessed by Lee *et al.* [27]. In particular, the *in situ* synthesis of Fe_3_O_4_ was compared to the stepwise addition of the same copolymer to preformed iron oxide colloids. In both cases, covalent binding was achieved between the magnetite surface and the siloxane functionalities of the polymer hydrolyzing to silanol groups, while PEG more likely remained on the outside of the SPIONs. The cross-linking reaction of the silanol groups by self-condensation, promoted by heating, resulted in more stable coating layers. The hydrodynamic radii of SPIONs prepared with and without copolymer in the NP synthesis step were 16.0 ± 2.2 nm and 12.3 ± 1.2 nm, respectively, with narrow size distributions and core sizes in the range of 4–8 nm in both cases, consistent with a thicker layer of coating by the *in situ* preparation as compared to sequential addition. However, the SPIONs synthesized *in situ* had an *M*_s_ value of 65 emu/g Fe (equivalent to 45.5 emu/g Fe_3_O_4_), significantly lower than the 80 emu/g Fe (56 emu/g Fe_3_O_4_) value obtained for the post-synthesis coated SPIONs. This effect can be interpreted as reflecting the influence on crystallinity or magnetic order of the NPs of surface complexation by the silanol groups of the copolymer during the synthesis. However, unlike ligands such as carboxylic acids and polyethers, that can enter the “inner coordination sphere” of Fe^2+^ and Fe^3+^ cations, silanols mainly interact with the Fe-OH hydroxyls at the surface of the iron oxide nuclei already formed in the sol-gel process, *i.e.*, the “outer sphere” of the metal centers. The risk of magnetic properties decreases trough surface ligand or spin-canting effects is therefore lower than for strong ligands introduced during the synthesis. Furthermore, the SPIONs synthesized were found to allow the detection of tumors in *T*_2_-weighted MR images within 1 h, as a result of the accumulation of the nanomagnets at the tumor site. Protein uptake by the surface of these coated SPIONs was reported to be significantly lower than by Feridex™ I.V., a popular contrast agent, thereby confirming the stealth effect in blood plasma of the PEG component of the copolymer.

#### 3.3.4. Coprecipitation in Water in the Presence of Poly(ethylene oxide)-*Block*-Poly(Methacrylic Acid) (PEO-*b*-PMAA) DHBCs

**Figure 6:**
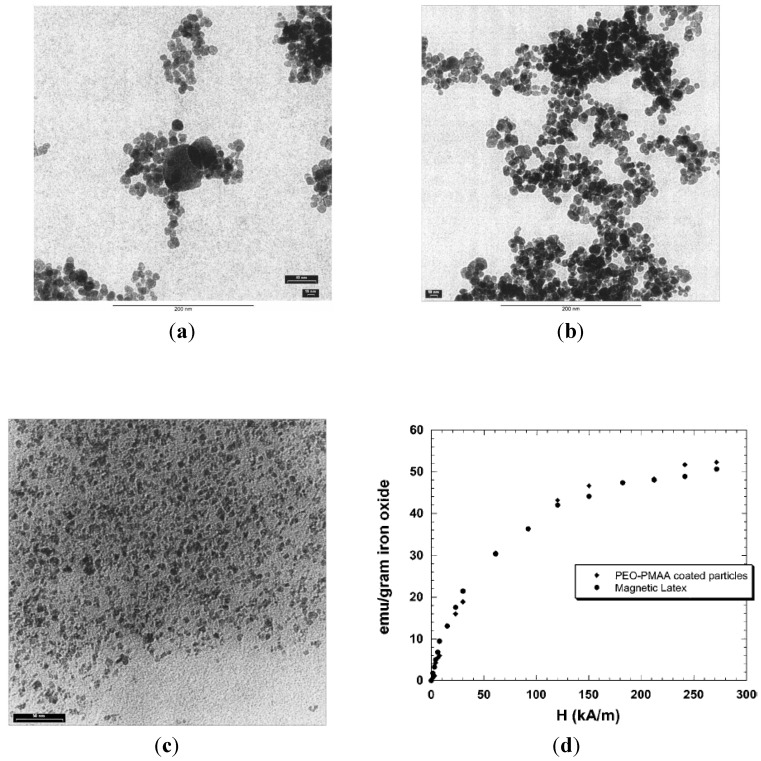
Transmission electron micrographs for magnetic iron oxide precipitated in: (**a**) water alone; (**b**) the presence of poly(ethylene oxide) (PEO) homopolymer; and (**c**) the presence of PEO-*b*-poly(methacrylic acid) (PMAA) block copolymer. Note that Figure 6c is at somewhat higher magnification than the others; (**d**) magnetization curves for the PEO-*b*-PMAA coated NPs and for P(HEMA-*co*-MMA) magnetic latexes obtained by inversion emulsion polymerization. Reprinted with permission from [28]. Copyright 2001 Elsevier.

In an attempt to confirm the roles of PEO-*b*-PMAA DHBC in directing the nucleation, controlling the growth, and sterically stabilizing the dispersions, a comparison was made among three cases: precipitation without polymer present, in the presence of “non-interacting” PEO, and in the presence of PEO-*b*-PMAA [28]. TEM images (Figure 6c) revealed that the particles coated with PEO-*b*-PMAA were much smaller (5 ± 4 nm) and more uniformly dispersed than those created using either PEO homopolymer or without polymer, highlighting the decisive role of carboxylic acid groups in the PMAA block in promoting nucleation and inhibiting growth of the magnetic iron oxide particles. Although crystallinity was confirmed by powder XRD (PXRD) analysis, the magnetite and maghemite phases could not be distinguished by that technique because these two phases have the same spinel structure and their XRD peaks positions are quite close to each other. The PEO-*b*-PMAA-coated oxide exhibited substantial magnetism, as seen on Figure 6d, with a magnetization of 50 emu/g at a field strength *H* = 275 kA/m or *B* = 0.34 T), from which we can infer a saturation level around 53 emu/g (so the value of 60 emu/g given by the authors appears a little over-estimated). The hydrophilic layer covering the particles facilitated their re-dispersion in hydrophilic mixtures of hydroxyethyl methacrylate and methacrylic acid monomers, before evaporation of the water. The waxy solid obtained was re-dispersed in an oil phase (decane) via sonication with poly(ethylene-*co*-butylene)-*block*-poly(ethylene oxide) (PBO-*b*-PEO) as emulsifier. Inverse emulsion polymerization of this sol yielded magnetic poly(hydroxyethyl methacrylate-*co*-methacrylic acid) P(HEMA-*co*-MMA) latexes with sizes broadly distributed from 35 nm to 250 nm, with the same superparamagnetic properties as the initial magnetic core-shell NPs, but also a lower overall magnetization (10 emu/g latex) corresponding to 18 wt% of iron oxide in the polymer matrix.

#### 3.3.5. Poly(Ethylene Glycol)-*Block*-Poly(Aspartic Acid) (PEG-*b*-PAsp) Leading to Akaganeite Rods

**Figure 7:**
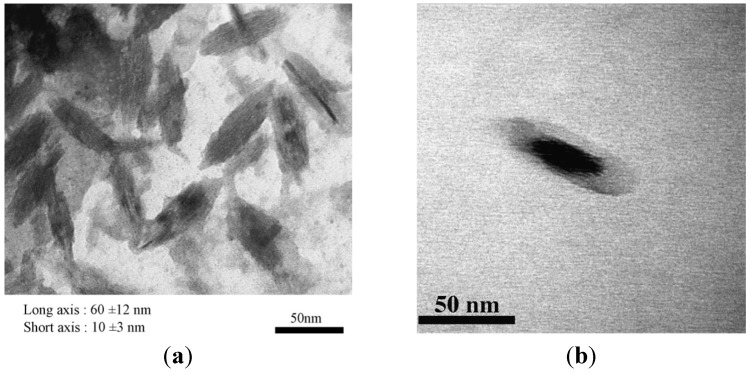
Energy filtering transmission electron microscopy (EFTEM) zero-loss images of: (**a**) β-FeOOH NPs; and (**b**) poly(ethylene glycol)-*block*-poly(aspartic acid) (PEG-*b*-PAsp)-coated β-FeOOH NPs. Reprinted with permission from [29]. Copyright 2007 Elsevier B.V.

In an effort to vary the chemical nature of the anchoring block as a polypeptide, while retaining PEG as stabilizing moiety, Kumagai [29] prepared coordination complexes between FeCl_3_ and PEG-*b*-PAsp in distilled water. Incubation of the mixture at 50 °C resulted in substantially homogeneous needle-shaped alkaganeite β-FeOOH particles, approximately 60 nm in length and 10 nm in width. Energy filtering transmission electron microscopy (EFTEM) zero-loss images revealed that the thickness of the PEG layer on the IONPs was 10–15 nm approximately (Figure 7). The anchoring interactions of the carboxylic groups with the β-FeOOH particles were confirmed using FTIR spectroscopy, with the successful resolution of simultaneous hydrogen and coordination bonding (complexation) interactions. However, a control experiment without polymer also led to needle-like oxo-hydroxides. The formation of the needles may thus be more likely ascribed to the physical conditions used (e.g., pH, salts, *etc.*) rather than to a templating effect of the polymer.

#### 3.3.6. Coprecipitation in Water with Poly(Ethylene Oxide)-*Block*-Poly(Acrylic Acid) (PEO-*b*-PAA)

**Figure 8:**
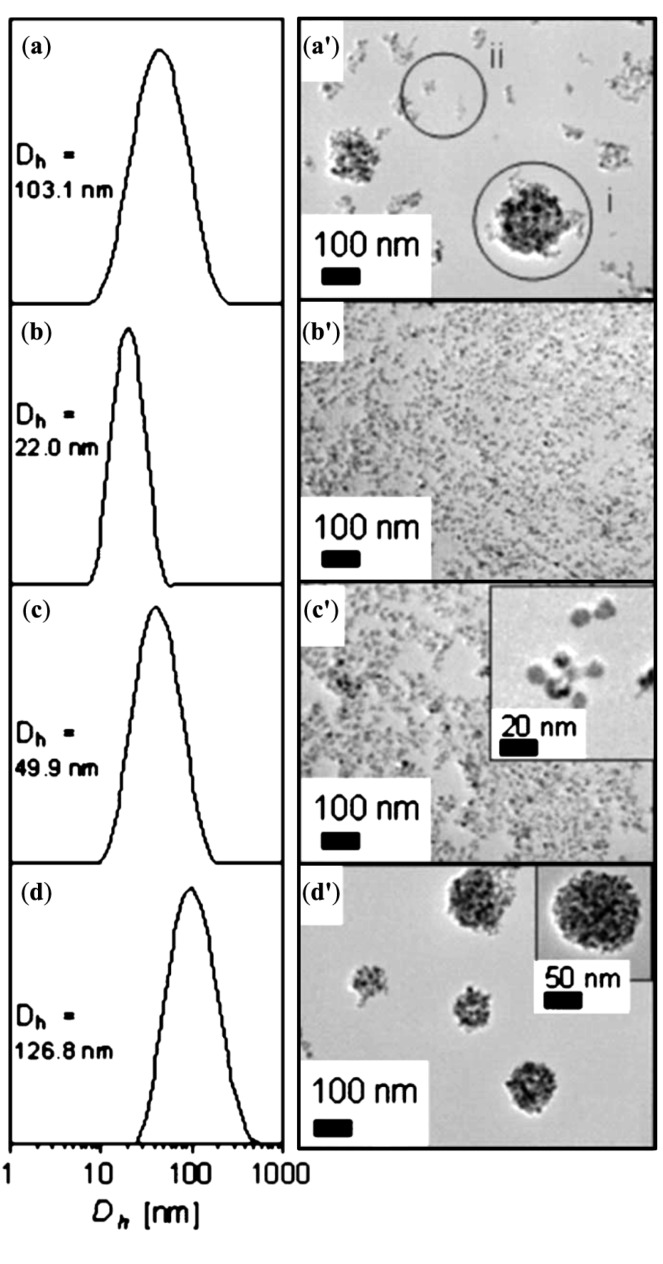
Size distribution: (**a**–**d**) by dynamic light scattering (DLS); and (**a'**–**d'**) from TEM images. (**a**,**a'**) the initial suspension after coprecipitation in presence of poly(ethylene oxide)-*block*-poly(acrylic acid) (PEO-*b*-PAA); (**b**,**b'**) the non-captured particles; (**c**,**c'**) the bleed-off samples; and (**d**,**d'**) the captured clusters. Reprinted with permission from [30]. Copyright 2009 Elsevier B.V.

In a study by Hatton *et al.* [30], the use of PEO-*b*-PAA in an aqueous coprecipitation route followed a size sorting step using a high-gradient magnetic separator (HGMS) yielded MNPs clusters with sizes larger than 100 nm, containing several particles with average core sizes of 9.5 nm. Both the bleed-off and captured clusters in the magnetic separator were superparamagnetic (*M*_s_ = 62.9 emu/g for the latter sample), thus indicating a single domain structure for the particles. The iron-to-polymer loading ratio (expressed as the molar ratio of the Fe^3+/2+^ cations to the polymer carboxylates) was varied, thus changing the polymer coverage of the MNPs. These results suggested that the formation of large clusters was induced by the limited amount of polymer added. Indeed, when a large excess of copolymer was used, the MNPs clusters obtained became small enough to pass though the magnetic separator without being arrested. This HGMS method is thus very interesting to separate constituents such as free DHBC and individually dispersed MNPs from clusters with a relatively well-controlled spherical morphology and a finite size, as shown in Figure 8.

#### 3.3.7. Deuterated Poly(Norbornene) (PNOR) Block Copolymers

**Figure 9:**
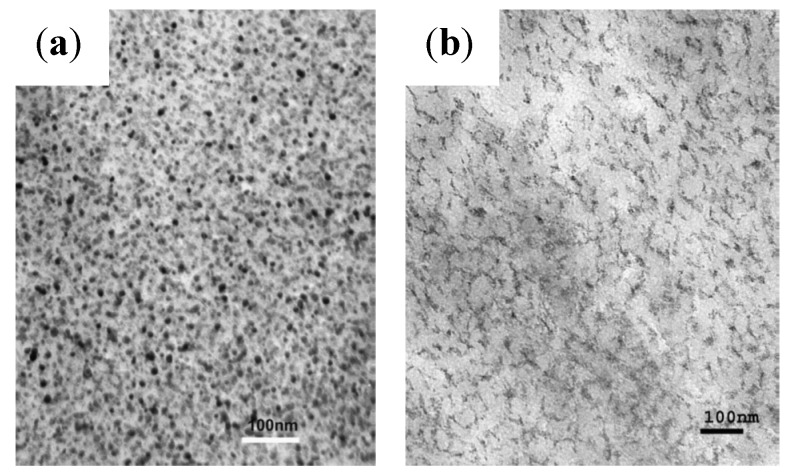
Morphology of poly(norbornene-*block*-deuterated norbornene dicarboxylic acid) loaded with iron oxide NPs (IONPs) at: (**a**) Ф_PNOR/PNORCOOH_ = 0.64/0.36 (disordered spheres); and (**b**) Ф_PNOR/PNORCOOH_ = 0.40/0.60 (interconnected spheres). PNOR: poly(norbornene); and PNORCOOH: poly(norbornene dicarboxylic acid). Reprinted with permission from [31]. Copyright 2005 Elsevier Ltd.

PNOR is a rubbery material with interesting properties, such as the ability to absorb up to 10 times its own weight of hydrocarbons while still retaining its high tear strength and vibration damping ability. Amphiphilic block copolymers of this material, suitable as templates for loading and co-precipitating iron salts, were obtained by the sequential ring-opening metathesis polymerization (ROMP) of norbornene and deuterated norbornene dicarboxylic acid (to provide better contrast between the two blocks in small-angle neutron scattering (SANS) experiments). The sodium salt form of poly(norbornene)-*block*-poly(norbornene dicarboxylic acid) (PNOR-*b*-PNORCOOH) copolymer was dissolved in a THF solution, to achieve ion exchange between Fe^3+^ and the Na^+^ ions of the carboxylate block. Thin films prepared from this solution were then soaked in NaOH solutions to produce γ-Fe_2_O_3_ particles [31]. Variations in the constituent block volume fraction ratio (Ф_PNOR/PNORCOOH_), while ensuring a constant 1:1 ratio of COOH/Fe^3+^, led to changes in the morphology and characteristic distances for the microphase-separated block copolymer that were correlated with the size and the mean inter-particle distance between the MNPs (as assessed by both SANS and the *T*_B_ measured by magnetometry). Starting from 10.4 nm diameter disordered spherical particles at Ф_PNOR/PNORCOOH_ = 0.64/0.36 (Figure 9a), the samples changed to interconnected particles with 16 nm diameter at Ф_PNOR/PNORCOOH_ = 0.50/0.50 and 0.40/0.60 (Figure 9b), thus suggesting the possibility of controlling the nucleating process through the separation of the iron-doped hydrophilic blocks by the hydrophobic walls between the domains. The *d*-spacing between the metal oxide NPs measured by TEM (e.g., 62 nm for 0.64/0.36) was consistent with the correlation distance obtained from SANS analysis (e.g., 53 nm for 0.64/0.36), which proved the success of using microphase-separated domains as template for the synthesis of IONPs. The ratio *d*/(2*R*), where *d* is the average distance between the particles given by SANS and 2*R* is the individual particle diameter from TEM, was found to be inversely related to the *T*_B_ of interconnected NPs. The sample at Ф_PNOR/PNORCOOH_ = 0.40/0.60, with the largest hydrophilic block, lead to the lowest *d*/(2*R*) ratio, and thus the highest *T*_B_ (115 K) and the highest *M*_s_ (76 emu/g at 300 K), in agreement with the largest magnetic moments and magnetic dipolar interactions between them.

**Figure 10:**
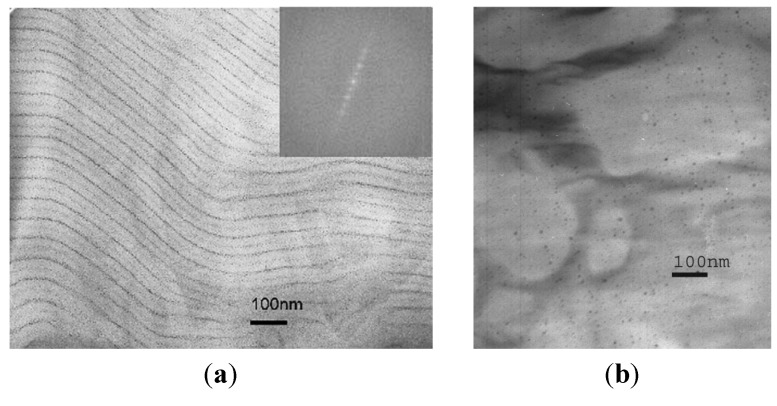
Electron micrographs for poly(norbornene methanol)**/**poly(norbornene dicarboxylic acid) (PNORMEOH/PNORCOOH) diblock copolymer: (**a**) stained with I_2_ vapor; and (**b**) doped with iron oxide by submerging a thin film in FeCl_3_ solution. Reprinted with permission from [32]. Copyright 2006 Elsevier Ltd.

The same *in situ* synthesis strategy was applied to a DHBC template of poly(norbornene methanol)-*block*-poly(norbornene dicarboxylic acid) (PNORMEOH-*b*-PNORCOOH) [32], resulting in a lamellar geometry with IONPs confined within the lamellar domains. Electron micrographs (with iodine staining) revealed that 6 nm average diameter γ-Fe_2_O_3_ particles decorated the lamellar structure of the copolymer in thin film samples, appearing in the images as thin lines of NPs, the spacing between the lines being 80 ± 10 nm (Figure 10).

#### 3.3.8. Poly(Styrene Sulfonate-*Alt*-maleic Acid) (PSS-*Alt*-MA) Shell with Subsequent Cross-Linking

The nucleating effects for *in situ* coprecipitation of amphiphilic PSS-*alt*-MA and of hydrophilic PAA were nicely demonstrated by Yoon *et al.* [33]. The coprecipitation was followed by cross-linking of the polymers with 1,6-hexanediamine, to prevent subsequent desorption of the polymer shell from the surface of the as synthesized IONPs and to reach a good colloidal state through electro-steric stabilization. The zeta potential remained highly negative after cross-linking (Table 3), even at high salt concentrations (e.g., 8%) added to reduce the thickness of the electric double layer thus weakening the electrostatic repulsions. The sub-100-nm IONPs clusters obtained were therefore stabilized by a balance of van der Waals and electrostatic interactions. The primary, highly crystalline IONPs exhibited superparamagnetism without hysteresis and an excellent *M*_s_ *ca*. 90 emu/g_Fe_, approaching the theoretical magnetization value for magnetite (92–100 emu/g_Fe3O4_, equivalent to 127–138 emu/g_Fe_), suggesting that the stabilizer did not lower the magnetization. These results suggest good stabilizing effects of PAA and PSS-*alt*-MA, while not demonstrating a true template effect since the MNPs were still polydisperse. 

**Table 3:** Zeta potential and average hydrodynamic diameters of poly(acrylic acid) (PAA) or poly(styrene sulfonate-*alt*-maleic acid) (PSS-*alt*-MA)-coated IONPs with different cross-linking densities for an iron oxide concentration of 0.14 wt% at pH 8. Adapted with permission from [33]. Copyright 2011 American Chemical Society.

Cross-linking (%)	Hydrodynamic diameter (DLS) (nm)	Zeta potential (mV)
PSS-*alt*-MA before cross-linking	44 ± 9	−48.6 ± 4.7
PAA before cross-linking	83 ± 2	−39.7 ± 1.5
PAA, 12.5% cross-linking	69 ± 17	−48.6 ± 1.1
PAA, 50% cross-linking	126 ± 9	−44.3 ± 2.2
PAA, 12.5% cross-linking	77 ± 16	−47.1 ± 2.2
PAA, 100% cross-linking	91 ± 2	−38.3 ± 3.0

#### 3.3.9. Brush Linear Poly(oligo(ethylene Glycol) Methacrylate-*co*-methacrylic Acid) P(OEGMA-*co*-MAA) as Nucleating and Stabilizing Ligand

Instead than relying upon the same copolymer block for both nucleation control and stabilization of the MNPs, Lutz *et al.* [34] developed back in 2006 the idea of using a double hydrophilic brush-linear block copolymer in which each block played a different, specific task. The coordinating ability of methacrylic acid units and the stabilizing capability of oligo(ethylene glycol) segments were examined in a coprecipitation process of iron ions, using hydrophilic P(OEGMA*-co-*MAA). The hydrodynamic diameter of the MNPs obtained was readily tuned in the range of 10–24 nm (Z-average values, polydispersity indices around 0.2) by variation of the initial polymer amount used as compared to the iron salts (from 1:1 to 0.42:1 wt:wt), thus also tuning their zeta potential from −14 mV to −7 mV. The 10 nm hydrodynamic diameter NPs were revealed as nearly mono-disperse 7 nm particles in the TEM micrographs. As evidenced by TEM the NPs had a quasi-spherical topology, in contrast to the “rock-like” shapes often obtained in coprecipitation methods. The stabilizing chain density was estimated as being relatively constantly in the range of 0.2–0.3 chains/nm^2^ in all cases, which could explain the correlation found between the size of the NPs and the polymer amount used in the reaction. Moreover, the colloidal stability of the ferrofluids in physiological media, enhanced by POEGMA block, allowed their *in vivo* use as MR imaging contrast agents.

#### 3.3.10. Coprecipitation with DHBCs of Poly(oligoethylene Glycol Acrylate) (POEGA) and Different Binding Blocks 

**Figure 11:**
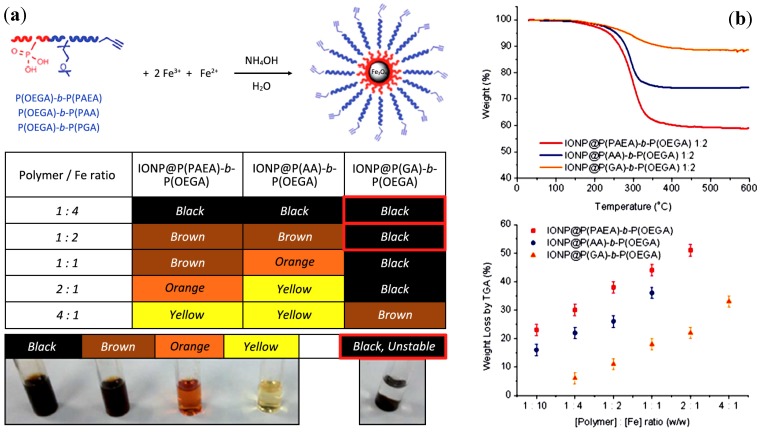
(**a**) *In situ* coprecipitation of iron oxide NPs (IONPs) at different polymer-to-Fe ratios; and (**b**) polymer content measured by thermogravimetric analysis (TGA) for IONPs coated with different anchoring groups at a 1:2 [Polymer]:[Fe] weight concentration ratio. Reprinted with permission from [35]. Copyright 2014 Royal Society of Chemistry.

The groups of Boyer and Davis described the synthesis by the xanthate reversible addition-fragmentation chain-transfer (RAFT/MADIX) technique of DHBCs composed of the same repelling block POEGA and anchoring blocks with either phosphonic acid ethyl acrylate (PAEA), carboxylic acid (PAA), or glycerol acrylate anchoring moieties in different copolymers [35]. These DHBCs were used for the *in situ* synthesis of IONPs by alkaline coprecipitation. The expected binding strength PAEA > PAA > PGA correlated nicely with the iron oxide loading, that also increased linearly with the feed-weight ratio of the iron salts (Figure 11b). The size of the MNPs measured both by TEM and XRD followed the reverse order (PAEA < PAA < PGA) for a given polymer-iron feed ratio, *i.e.*, the stronger the affinity for iron of the ligand, the smaller the magnetic core size. The size also decreased for increasing polymer contents (Table 4). All these observations confirmed a high degree of control over the nucleation and growth of the IONPs achieved with the copolymer template. The final products consisted in hybrid magnetic multi-core clusters (as seen on the TEM images), exhibiting good colloidal stability in biological buffers and high *T*_2_ relaxivity, which had the authors envision their use for *in vivo* MRI.

**Table 4:** Summary of weight loss TGA results and particle size (*d*_TEM_ and *d*_XRD_) of polymer-coated IONPs at different polymer-to-iron ratios [35]. PAEA: phosphonic acid ethyl acrylate. Copyright 2014 Royal Society of Chemistry.

Hybrid magnetic core-shells	[Polymer]:[Fe] weight ratio	Wt loss TGA	*d*_TEM_ (nm)	*d*_XRD_ (nm)	Grafting density (nm^−2^)
IONP@P(PAEA)-*b*-P(OEGA)	1:10	23%	10.45	9.83	0.083 ± 0.005
	1:4	30%	9.80	9.10	0.124 ±0.003
	1:2	38%	9.40	8.52	0.168 ± 0.008
	1:1	44%	8.64	7.32	0.192 ± 0.01
	2:1	51%	-	-	-
IONP@P(AA)-*b*-P(OEGA)	1:10	16%	10.25	10.09	0.065 ± 0.001
	1:4	22%	8.91	8.75	0.084 ± 0.003
	1:2	26%	7.83	7.44	0.091 ± 0.003
	1:1	36%	-	-	-
IONP@P(GA)-*b*-P(OEGA)	1:4	6%	12.39	11.47	0.023 ± 0.001
	1:2	11%	10.24	10.62	0.039 ± 0.001
	1:1	18%	9.14	9.78	0.063 ± 0.002
	2:1	22%	8.65	8.41	0.073 ± 0.001
	4:1	33%	-	-	-

#### 3.3.11. Spherical Micelles Loaded with IONPs

**Figure 12:**
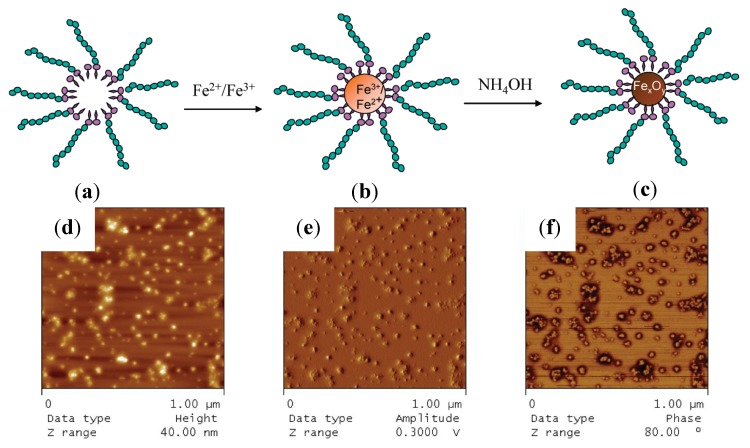
Strategy for the preparation of stabilized magnetic IONPs in aqueous media (**top**): (**a**) micelle formation by PEGMA*_x_*-*b*-AEMA*_y_* diblock copolymers in water; (**b**) addition of the Fe^3+^/Fe^2+^ mixture to the micellar solution, leading to complexation of the iron salts by the β-ketoester units inside the micellar core; and (**c**) transformation of the iron salt “precursor” into IONPs inside the micellar core upon addition of NH_4_OH solution; atomic force microscopy (AFM) images of the block copolymer micelles loaded with IONPs (**bottom**): (**d**) height image; (**e**) amplitude image; and (**f**) phase image. Reprinted with permission from [36]. Copyright 2009 American Chemical Society.

In the same context, amphiphilic block copolymers were also used as templates by introducing bidentate moieties, such as in poly[2-(acetoacetoxy) ethyl] methacrylate (PAEMA), to promote the nucleation of iron oxide. Papaphilippou *et al.* [36] investigated the use of this hydrophobic polymer in a copolymer with PEGMA as a hydrophilic and biocompatible second block, but also having a LCST near 60 °C. Highly stable hybrid micelles, visualized by atomic force microscopy (AFM) as spherical micelles loaded with IONPs (Figure 12), were obtained at various precursor salt concentrations. The maximum amount of iron that could be encapsulated without precipitation was unfortunately not determined. A plateau magnetization *M*_s_ of 300 A/m was reached for the PEGMA_70_-*b*-AEMA_16_-coated MNPs at a 3:1 [Fe^3+^]/[AEMA] molar ratio, but the specific magnetization cannot be calculated for this system since the corresponding iron concentration used was not specified. *In vitro* biocompatibility and macrophage uptake tests were also conducted and were encouraging in terms of toxicity and the minimization of recognition and phagocytosis.

As an intermediate case between (fully hydrophilic) DHBCs and amphiphilic block copolymers, graft polymers of composition PAA-*g*-PEO/PPO (PPO: poly(propylene oxide)) were studied by the group of Hatton [37]. In analogy with the commercial Pluronics^®^ systems (PEO-*b*-PPO-*b*-PEO), the idea was to vary the hydration level of the micelles by tuning their PEO/PPO content while using the PAA backbone to complex with Fe^2+^/Fe^3+^ ions. After alkaline coprecipitation, the water-based magnetic fluids obtained consisted of *ca*. 7.3 nm (TEM) magnetite NPs decorating the amphiphilic graft copolymer micelles. The magnetic core radii obtained by TEM and neutron scattering analyses exhibited broad size distributions and irregular particle shapes, however. Therefore the self-assembly of such copolymers with iron oxide precursors does not appear to provide a sufficient template effect for the synthesis of well-calibrated MNPs, and better controlled nanostructures need to be explored as reactors.

## 4. Synthesis Templated by Preformed Structures

Rather than using individual polymer chains and inducing their self-assembly in the presence of metallic salts, loading can be achieved by exposing preformed self-assembled structures to these precursors. This strategy is expected to minimize changes in morphology or size dispersity, but the ability of the metallic precursors to reach the anchoring moieties might be affected. Consequently, several studies aimed at optimizing the conditions for the *in situ* synthesis of MNPs in individual nanosized, well-defined objects.

### 4.1. Microemulsions in an Organic Solvent

The size, shape, morphology and physical properties of *in situ* synthesized MNPs are not only strongly influenced by the nature of the templates, but also by the methods used to load the precursors and create the MNPs. In emulsion methods, the nanosized droplets of water-in-oil microemulsions appear ideal as reactors inside which NPs can be synthesized, with a monodispersed distribution of diameters below 20 nm. The results obtained in this approach depend on the type of amphiphilic species used to stabilize the microemulsion.

#### 4.1.1. Chitosan Shells in Fe^2+^ Microemulsions with Triton^®^-X

**Figure 13:**
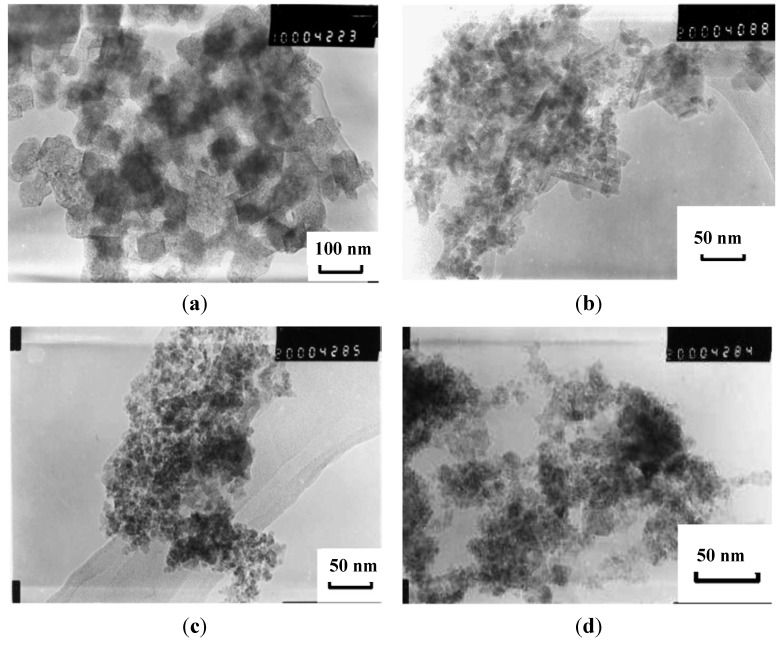
TEM of magnetic NPs (MNPs) obtained with: (**a**) molecular weight (MW) = 5.0 × 10^5^ g∙mol^−1^ non cross-linked chitosan; (**b**) cross-linked; (**c**) MW = 1.0 × 10^5^ g∙mol^−1^ non cross-linked chitosan; (**d**) cross-linked. Reprinted with permission from [38]. Copyright 2006 Elsevier B.V.

Due to the presence of numerous amine and hydroxyl groups, chitosan, a derivative of chitin with an acetylation level below 60%, has been considered as a template for loading metallic ions. Combined with other useful properties such as being non-toxic, hydrophilic, biocompatible, biodegradable and anti-bacterial, chitosan can be useful to shorten the gap between the synthesis of the MNPs and their biomedical applications. In research combining the advantages of microemulsion systems with the benefits of chitosan templates, Zhi *et al.* [38] proposed a route in which NaOH solution was quickly added to an microemulsion system of cyclohexane, *n*-hexanol, HCl, chitosan, and a ferrous salt, stabilized by the Triton^®^-X 100 surfactant with controlled exposure to oxygen. The type of iron oxide obtained in the NPs was efficiently controlled by the amount of oxygen in the N_2_ gas added during the synthesis, with 0.05% of oxygen yielding 60–80 nm spherical chitosan particles encapsulating cubic-shaped Fe_3_O_4_ cores (Figure 13). Glutaraldehyde (GDA) crosslinking of the magnetic chitosan NPs was suggested as a method to reduce the diameter of the obtained particles in the range of 10–50 nm without affecting the Fe_3_O_4_ cores. *M*_s_ values of 11.15 emu/g were reported for the composite NPs, in accordance with their iron oxide content. 

#### 4.1.2. Quaternary Microemulsions to Produce Aligned Spinel CoFe_2_O_4_ Nanorods

**Figure 14:**
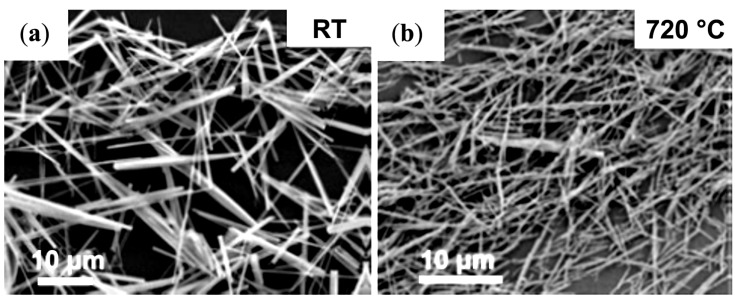
Scanning electron microscope (SEM) images for: (**a**) as-synthesized CoFe_2_(C_2_O_4_)_3_ suspension after coprecipitation in a microemulsion; and (**b**) CoFe_2_O_4_ rods annealed at 720 °C. Reprinted with permission from [39]. Copyright 2005 Wiley-VCH.

**Figure 15:**
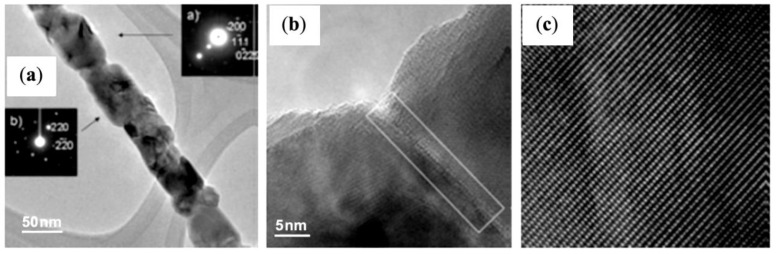
(**a**) TEM image of an individual CoFe_2_O_4_ nanorod annealed at 720 °C; the insets illustrate SAED patterns acquired from two individual nanocrystals of size about 80 nm. Insert (**a**) shows the indexed diffraction pattern for the *fcc* crystals in the [011] beam direction, and insert (**b**) in the [001] beam direction; (**b**) high resolution transmission electron microscopy (HRTEM) image showing a grain boundary between two CoFe_2_O_4_ nanocrystals; and (**c**) HRTEM image for a CoFe_2_O_4_ nanocrystal. Reprinted with permission from [39]. Copyright 2005 Wiley-VCH.

Quaternary microemulsions of cetyl trimethylammonium bromide (CTAB), water, cyclohexane and pentanol were used as microreactors for the coprecipitation of Co^2+^, Fe^2+^ and oxalate anions, followed by the decomposition at high temperature of CoFe_2_(C_2_O_4_)_3_ (as “sacrificial template”) and oxidation of Fe^2+^ into Fe^3+^ to produce spinel CoFe_2_O_4_ nanorods [39]. The calcination treatment was indispensable to convert the amorphous nanorods into crystalline nanorods while maintaining control over their dimensions (50–100 nm in diameter, several micrometers in length), by stimulating internal nucleation and crystallization, in analogy to the natural petrification process which requires centuries to complete. scanning electron microscope (SEM) images highlighted the overall rod-like shape of the particles (Figure 14), and TEM images revealed the morphological transformation and the ripening of the rod-like particles upon heating, leading to the final state with individual CoFe_2_O_4_ crystals linearly arranged along the annealed nanorods (Figure 15a). The crystallographic alignment of the FeCo_2_O_4_ nanocrystals became visible after calcination in the high resolution TEM images as atomic planes (Figure 15c) and grain boundaries between the interconnected nanocrystals (Figure 15b). After annealing, the nanorods also exhibited magnetic *H*_c_ with a Curie temperature well above room temperature. Regarding their formation mechanism, the authors noted that the direct calcination of a co-precipitated CoFe_2_(C_2_O_4_)_3_ suspension in the absence of the microemulsion template did not lead to long crystalline anisotropic magnetic structures. Therefore an “oriented attachment” mechanism between the inorganic nuclei growing through the organic template was proposed. The ionic nature of the CTAB surfactant may also have played a role at the beginning of the calcination process, by forming a molten salt also favoring oriented nucleation. 

### 4.2. Spherical Micelles in Water

Templates with predetermined sizes and structures are good candidates to orient the nucleation process, as they can be designed with an appropriate anchoring block, carry highly stabilizing units, and optionally a cross-linkable segment providing additional versatility.

#### 4.2.1. Triblock Polyisoprene-*Block*-poly(2-cinnamoylethyl Methacrylate)-*Block*-Poly(*Tert-*Butyl Acrylate) (PI-*b*-PCEMA-*b*-PtBA) Copolymer Hollow Nanospheres

**Figure 16:**
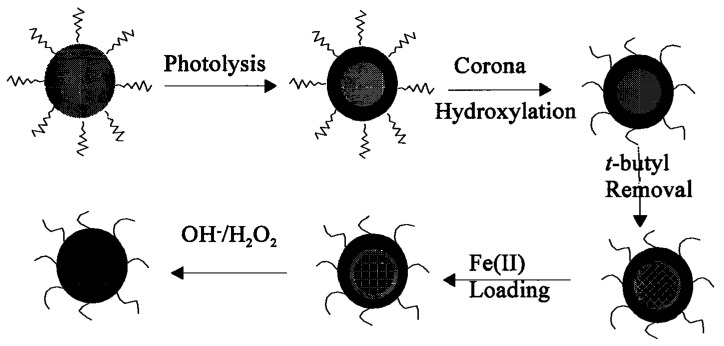
Polyisoprene-*block*-poly(2-cinnamoylethyl methacrylate)-*block*-poly(*tert-*butyl acrylate) (PI-*b*-PCEMA-*b*-P*t*BA) as template: Photolysis cross-links the PCEMA shell (gray to black); the PI corona chains are made water-soluble by hydroxylating the double bonds (wavy lines to free-hand lines); the core is made inorganic-compatible by removing the *tert*-butyl groups (light gray to gridded pattern). Soaking the nanospheres in aqueous FeCl_2_ leads to proton exchange (slanted to vertical grids) and the Fe^2+^ ions are precipitated (NaOH) and oxidized (H_2_O_2_) to yield cubic γ-Fe_2_O_3_ magnetic particles (last step). Adapted with permission from [40]. Copyright 2000 American Chemical Society.

**Figure 17:**
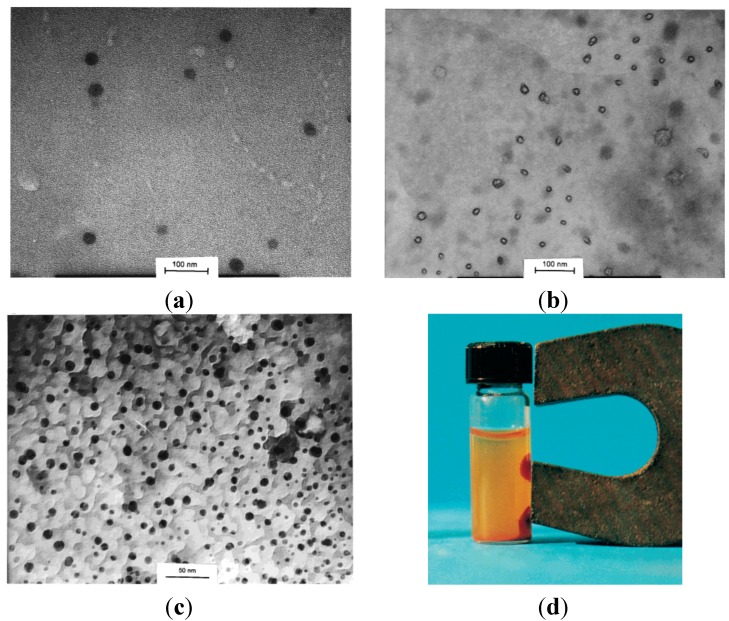
TEM images for PI-*b*-PCEMA-*b*-PtBA nanospheres at each step of the synthesis: (**a**) after PCEMA cross-linking and PI hydroxylation (stained with OsO_4_); (**b**) after removal of the *tert*-butyl groups (stained with OsO_4_); (**c**) after Fe_2_O_3_ loading (no staining); and (**d**) attraction by a magnet. Adapted with permission from [40]. Copyright 2000 American Chemical Society.

At first, spherical micelles with a structure locked by photo-cross-linking were prepared using a triblock copolymer PI-*b*-PCEMA-*b*-P*t*BA, in which PI forms the corona, PCEMA a solvent-insoluble shell, and P*t*BA the hydrolysable core [40]. Then, hydroxylation of the PI corona and cleavage of the *tert-*butyl groups to convert PtBA to PAA enabled the coordination of Fe^2+^ ions in the template. Finally, alkaline oxidation with NaOH and H_2_O_2_ yielded water-soluble Fe_2_O_3_-impregnated nanospheres. This multi-step pathway is represented schematically on Figure 16. The diameter of the polymeric template (90 nm) did not change after the *tert-*butyl removal step (as seen by comparison of Figure 17b with Figure 17a), indicating the benefits of using templates with a predetermined size, shape and morphology. Inorganic cores with diameters ranging from 4 nm to 16 nm were visible on TEM images (Figure 17c). The Bragg peaks detected in selected area electron diffraction (SAED) experiments were matched with the Miller indices of the maghemite (γ-Fe_2_O_3_) phase. Therefore, such templates with a cross-linked polymer shell surrounding a porous polymer core coordinating ferrous precursors were efficient as templates for the preparation of IONPs, although further studies were suggested to control the size distribution of the magnetic cores.

#### 4.2.2. PEG-*block*-Poly(4-Vinylbenzylphosphonate) (PEG-*b*-PVBP) Micelles

**Figure 18:**
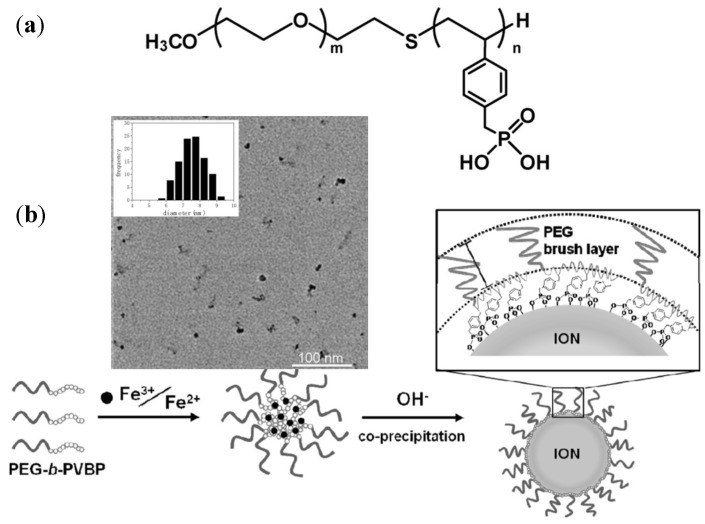
(**a**) Chemical structure of PEG-*b*-PVBP and schematic illustration of the proposed morphology of the pegylated IONPs (PIONs); and (**b**) TEM image of the PIONs and their size distribution as inset. PVBP: poly(4-vinylbenzylphosphonate). Adapted with permission from [41]. Copyright 2011 Elsevier B.V.

Among the papers collected for this review, a small number concerned the density of surface coatings of pegylated IONPs (PIONs) for *in vivo* applications such as MRI. A recent study by Ujiie *et al.* [41] utilized PEG-*b*-PVBP block copolymers for *in situ* alkaline coprecipitation (Figure 18). A correlation between the (PEG-*b*-PVBP)/(iron salt) feed ratio and the surface density of PEG chains in PEG-PIONs was revealed, suggesting an optimal ratio to obtain sufficiently small particles with a high PEG chains density. Interestingly, while the core sizes measured by TEM (≈7.7 nm) were typical for the aqueous coprecipitation route, they were unrelated to the feed ratios used. Thus although a “template effect with micelle-like aggregates” was invoked, the main interest of this method is for the preparation of individually dispersed MNPs stable in biological buffers rather than achieving precise control over the size of the magnetic cores. It is worth noting that the phosphonate groups significantly contributed to the incorporation efficiency of the block copolymer, due to its well-known binding ability for various metal oxides. The *R_h_* ≈ 30 nm observed for the micelle-like aggregates before the ammonia addition was considered as reflecting a “templating effect” for metallic nucleation. This is the main reason why this study is reported in the preformed structures section instead of the block-copolymer section.

#### 4.2.3. Multiarm Star-Like Amphiphilic or DHBCs

**Figure 19:**
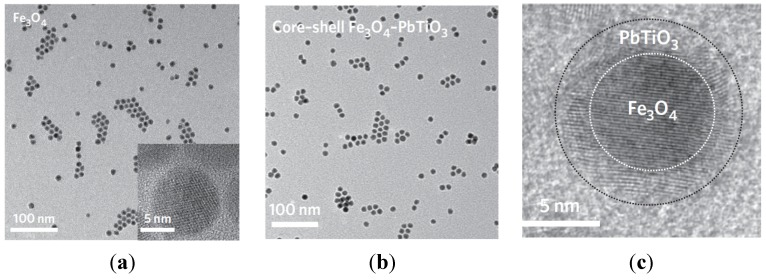
(**a**) Representative TEM images for Fe_3_O_4_NPs synthesized with star-like PAA-*b*-PS templates, *D*(Fe_3_O_4_) = 10.1 ± 0.5 nm; (**b**) TEM; and (**c**) HRTEM images of Fe_3_O_4_-PbTiO_3_ core-shell NPs formed with the star-like triblock copolymers nanoreactors. Reprinted with permission from [42]. Copyright 2013 Macmillan Publishers Limited.

**Figure 20:**
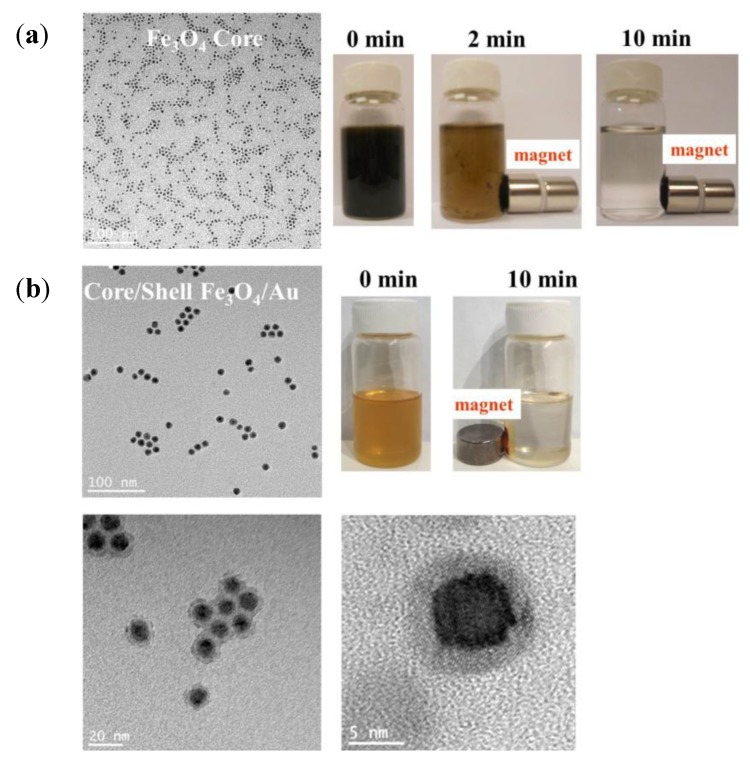
TEM, HRTEM and digital camera images for core/shell Fe_3_O_4_/Au NPs: (**a**) Fe_3_O_4_ core (*D* = 6.1 ± 0.3 nm); and (**b**) Fe_3_O_4_/Au core/shell NPs at different magnifications (Au shell thickness is 2.9 ± 0.2 nm). Fe_3_O_4_ appears dark in the center. The magnetic properties of Fe_3_O_4_ were retained, as evidenced by the response of the NP dispersion in toluene to a magnet (right panel in (**b**)). Reprinted with permission from [42]. Copyright 2013 Macmillan Publishers Limited.

For linear block copolymer templates, the size, shape, and the colloidal stability of the nanocrystals are greatly influenced by experimental conditions used (e.g., concentration, solvent, temperature, and pH). Multi-arm star-like unimolecular micelles derived from either amphiphilic or DHBCs were successfully employed to achieve better control over the size and shape of the MNPs. Starting from a multi-arm ATRP initiator (suitably functionalized β-cyclodextrin (CD)), the polymeric blocks (P*t*BA or P4VP) serving to bind the inorganic precursor were synthesized first. These star-like homopolymers we then coupled by “click” chemistry with either hydrophobic (PS) or hydrophilic polymeric (PEO) segments which imparted solubility to the micelles in either organic or aqueous environments. The FeCl_2_ and FeCl_3_ precursors were loaded in the hosts in appropriate solvents. After ammonium hydroxide addition, Fe_3_O_4_ NPs were produced with different sizes (6, 10 and 16 nm, e.g., Figure 19a and Figure 20a), depending on the of the PAA block derived from P*t*BA (4.5, 8.4 and 16.8 kDa). These exhibited superparamagnetic properties at 300 K without hysteresis, and *M*_s_ increasing with the NP size. The advantages of triblock structures (β-CD-P4VP-P*t*BA-PS) and (β-CD-P4VP-P*t*BA-PEO) were also highlighted by producing NPs with Fe_3_O_4_ core and PbTiO_3_ (Figure 19b,c) or Au shell (Figure 20b). More accurately, the magnetite NPs were encapsulated within the P4VP core, and the PbTiO_3_ or Au layers were grown within the PAA shell (derived from the P*t*BA block after the first encapsulation step), thus retaining the magnetic properties of the NPs. TEM imaging revealed a uniform size and narrow size distribution for the NPs, while the crystalline lattices were characterized by HRTEM, XRD and energy-dispersive X-ray spectroscopy (EDS) measurements. Hollow gold NPs were also reported, aside from the magnetic-gold core-shell systems.

This work undoubtedly evidences a strong templating effect in the *in situ* synthesis of inorganic NPs, in particular magnetic ones, using unimolecular star-like PAA-PEO micelles.

### 4.3. Cylindrical Multimolecular Micelles

**Figure 21:**
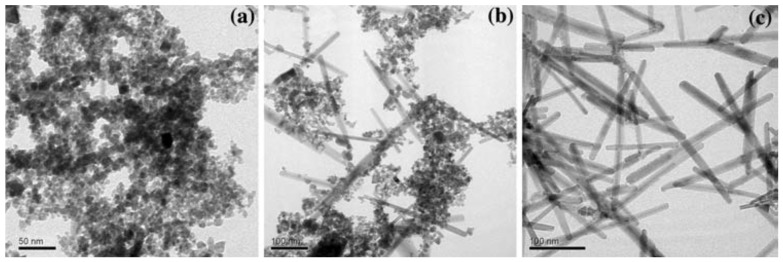
TEM images for samples prepared in alcohol/water mixtures with various volume ratios of alcohol to water: (**a**) 0:1; (**b**) 1:1; and (**c**) 5:1. Reprinted with permission from [43]. Copyright Springer Science Business Media, LLC 2008.

Different morphologies were obtained by self-assembly of commercial Pluronic^®^ F127 PEO-*b*-PPO-*b*-PEO triblock copolymers in water/alcohol mixtures, to serve as templates for iron ion loading by coprecipitation [43]. Surprisingly, as the alcohol content in the solvent mixture increased, a gradual phase transformation from 15 nm Fe_3_O_4_ NPs (Figure 21a) to uniform (*ca*. 20 nm diameter, 200–300 nm length) α-FeOOH (goethite) nanorods (Figure 21b) was observed. This shows that Pluronics^®^ can serve as structure-directing agents that control both the metal oxide mesoscale size and the crystalline structure. The decrease in *M*_s_ of the samples observed was in agreement with this phase transformation from the superparamagnetic (maghemite) to the anti-ferromagnetic state (goethite).

#### 4.3.1. Fe^3+^ Loading in Poly(Acrylic Acid)-*Graft*-Poly(*n*-Butyl Acrylate) Brush

**Figure 22:**
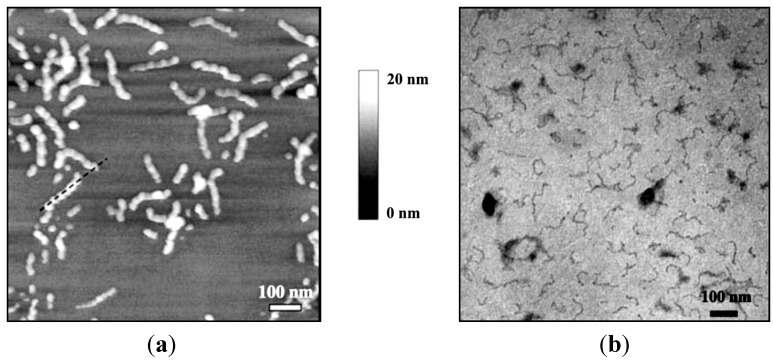
(**a**) AFM height image of the polychelate of Brush 1 and FeCl_3_ after dialysis; and (**b**) TEM image of uni-molecular polymer brush polychelate of FeCl_3_ (after dialysis). Adapted with permission from [44]. Copyright 2004 Springer-Verlag.

In an attempt to preparing hybrid magnetic unimolecular comb-like polymer micelles, Zhang and Muller reported the procedure of employing amphiphilic core-shell cylindrical polymer brushes with poly(acrylic acid)-*block*-poly(*n*-butyl acrylate) (PAA-*b*-PnBA) side chains to impregnate Fe^3+^ [44]. It was worth noting that in order to increase the rate and extent of iron ion uptake, COOH groups in PAA blocks were prior deprotonated by NaOH before the encapsulating step performed with an excess amount of FeCl_3_. The polychelate effect was proved by FTIR and ultraviolet-visible spectroscopy (UV-VIS) measurements, while the TEM and AFM images revealed the core of the unimolecular wormlike cylinder hybrids that have been mineralized by the ferric cations (Figure 22). However, the authors did not go further than the precursor loading, *i.e.*, they did not describe the alkaline coprecipitation *in situ*.

#### 4.3.2. Fe^3+/2+^ Loading and Precipitation in Poly[Poly(Ethylene Glycol) Methylether Acrylate]-*Graft*-Poly(Methacrylic Acid) (PPEGMEA-*g*-PMAA) Brush

**Figure 23:**
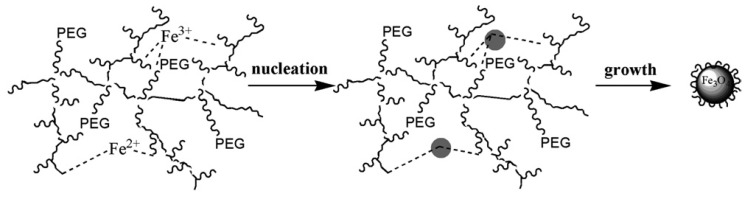
Synthesis of Fe_3_O_4_/polymer nano-composite in the presence of PPEGMEA-*g*-PMAA densely grafted double hydrophilic copolymer. PPEGMEA: poly(ethylene glycol) methylether acrylate. Reprinted with permission from [3]. Copyright 2008 Royal Society of Chemistry.

Both nucleating and stabilizing abilities were introduced into a densely grafted bottle-brush double hydrophilic copolymer constituted of two different types of hydrophilic segments, respectively a PMAA backbone, providing COOH groups for nucleation of Fe_3_O_4_ particles, and poly[poly(ethylene glycol) methylether acrylate] (PPEGMEA) side chains (Figure 23), introducing a shielding effect to prevent the unfavorable aggregation. Such template was investigated for an *in situ* coprecipitation process [3]. Narrow size distributed Fe_3_O_4_/polymer clusters with hydrodynamic sizes were 50–210 nm, carrying 7–15 nm diameter Fe_3_O_4_ crystals were obtained. The magnetic core sizes were efficiently tuned by varying the polymer/Fe_3_O_4_ feed ratios and the length of PMAA side chains. For example, the diameter of the MNPs increased from 8.5 nm to 9.5 nm and 10.4 nm when this ratio was varied from 1/1 to 1/2 and 1/3. Measurements on a VSM setup revealed the superparamagnetism of the hybrids at room temperature, with a concomitant increase of the *M*_s_ value from 48 emu/g, to 49 emu/g and 50 emu/g. Such dense polymeric combs were thus considered as influent templates to orient the distribution of sizes and to improve the magnetization properties of the Fe_3_O_4_ NPs.

#### 4.3.3. Poly(Glycerol Monoacrylate)-*Graft*-Poly(PEG Methyl Ether Acrylate) (PGA-*g*-PEG) Copolymers

In a detailed study [45] aimed at investigating the influence of the graft density *g* and MW of the graft polymer PGA-*g*-PEG on the average size and magnetic properties of MNPs, a possibility of changing the diameter of particles in 4–18 nm range by employing polymers with different grafting densities was suggested. It is understandable that as the hydroxyl groups in PGA block were the anchoring functionalities, the decreased feed ratio of the monomeric precursors of glycerol monoacrylate to the PEG methyl ether acrylate side chains in graft copolymerization resulted in the rise of the graft density *g*, yet reducing the coordinating capability of each individual graft polymer micelle, thus leading to the ascending particle sizes. This observation was confirmed by the data obtained from XRD measurement were the Bragg peaks widths were analyzed by the Scherrer equation: the average particle sizes were 4, 7, 9 and 18 nm, respectively, for *g* = 0.14, 0.23, 0.30 and 0.42. Interestingly, graft copolymers with varied MW (from 4.2 kD to 7.6 kD and 15 kD) bearing the same graft density *g* = 0.24 produced particles of similar size. The particles were superparamagnetic at room temperature with normalized *M*_s_ decreasing in the range of 30–42 emu/g with reducing particle sizes, due to the smaller domains crystallized.

#### 4.3.4. Poly(Ethylene Oxide)-*Graft*-Poly(Acrylic Acid) (PEO-*g*-PAA) Graft Copolymer

The four-arm graft copolymers PEO-*co*-ethoxyethyl glycidyl ether-*graft*-PAA [PEO*_x_*-*co*-Gly*_y_*-*g*-PAA*_z_*]_4_ were employed in the same coprecipitation process as reported above leading to highly dispersed narrow size distributed particles encapsulating 10–20 nm iron oxide monocrystals inside the polymer chains [46]. The TEM results were in good agreement with those from XRD analysis with the exception of the peak broadening which was assigned for the matrix constrain of the nanosized particles. The superparamagnetic properties were confirmed, and the calculated *M*_s_ was 56 emu/g Fe_3_O_4_. The size and the size distribution of hybrid NPs were tuned by changing the mass ratio of polymer compared to Fe_3_O_4_ and by varying the ratio of PAA/PEO segments in the graft copolymer (better template effect with smaller particles and narrower size distribution was reported with larger proportion of PEO segments). More importantly, star-shaped graft polymers with more confined structure exhibited better template effect as compared to linear graft copolymer consisting of similar components.

From the three preceding examples, which originate from different teams, one can conclude that despite some little changes of the chemical nature of the polymer segments, the graft polymer structures always induce a reproducible trend in the variation of the average size of the magnetite NPs synthesized by *in situ* coprecipitation. However, these studies may have overestimated the template effect, in the sense that the size distributions observed on TEM images are always broad, and do not correspond to true monodisperse samples.

### 4.4. Lamellar Films

#### Polystyrene-*Block*-Poly(2-Vinylpyridine) (PS-*b*-P2VP) Lamellae Hosting Pristine Iron Nanoparticles

**Figure 24:**
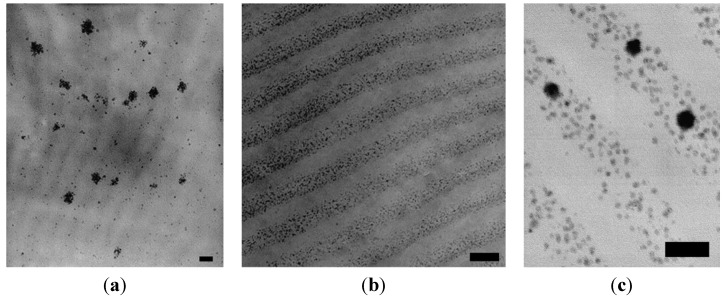
(**a**) TEM micrograph for a lamellar PS-*b*-P2VP copolymer containing 1.2 meq of iron/g P2VP; sample heated to 195 °C for 24 h, scale bar = 50 nm; (**b**) TEM micrograph for the same copolymer containing a total of 1.2 meq of Fe and Co/g P2VP, with an atomic ratio of Fe to Co of 80:20; sample heated to 161 °C for 24 h, scale bar = 100 nm; and (**c**) scanning transmission electron microscopy (STEM) micrograph of the copolymer containing a total of 1.2 meq of Fe and Co/g P2VP, atomic ratio of Fe to Co = 50:50; sample heated to 161 °C for 24 h; the atomic ratio of Fe:Co at the center of the large particles was 48.9:51.1 from energy dispersive X-ray spectroscopy (EDX) spectroscopy; scale bar = 50 nm. Reprinted with permission from [47] Copyright 2003 Elsevier B.V.

The complexation ability of vinylpyridine moieties was investigated by the groups of Cohen and Ross [47] not only in the form of linear homo- and copolymers as discussed above, but also in bulk films of PS-*b*-P2VP. Nucleation was induced by the thermal decomposition of Fe(CO)_5_ or Co_2_(CO)_8_. The impregnation of the organometallic complexes Fe(CO)_5_ and nickelocene in vacuum-dried films produced monodispersed iron, iron-cobalt, and cobalt-nickel metallic NPs residing within the P2VP domains, that were superparamagnetic at room temperature. The lamellar structure of these composites was shown in TEM micrographs as a “cluster of grapes” morphology of pristine metal particles that were monodispersed in size when the metallic feed ratio of Fe to Co was 100:0 (Figure 24a) or 80:20 (Figure 24b), and bimodal when increasing the Co content to 50:50 (Figure 24c) or 20:80. The magnetization values of the samples varied from 1.1 emu/g P2VP in the first two cases to 8.4 emu/g P2VP and 10.9 emu/g P2VP for the higher Co contents. The increase in *M*_s_ observed for higher Co contents was explained by the preference for cobalt atoms to remain at the surface of the particles to minimize their surface energy, which led to thinner layers and more walls (as shown by scanning transmission electron microscopy (STEM)) separating smaller particles with a larger surface-to-volume ratio. The smaller particles nucleated on the film were considered a dominant factor explaining the non-zero *H*_c_ observed at room temperature for some samples.

### 4.5. Hexagonal Ordered Films

#### 4.5.1. Monolayer Films of Polystyrene-*block*-Poly(4-Vinylpyridine) (PS-*b*-P4VP) Copolymer Micelles

**Figure 25:**
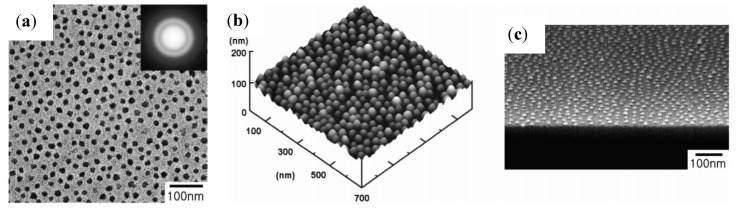
(**a**) TEM image for a self-assembled pattern of IONPs with hexagonal packing. The inset is a SAED pattern typical of γ-Fe_2_O_3_; (**b**) AFM image for the hexagonal pattern of IONPs on a silicon wafer; and (**c**) field emission-scanning electron microscopy (FE-SEM) image in tilt view. The molar ratio of FeCl_3_ precursor to 4-vinylpyridine was 0.5 in all cases. Reprinted with permission from [48]. Copyright 2005 American Chemical Society.

To create monolayer films where the particles were temporarily arranged in a hexagonal array, PS-*b*-P4VP micelles (formed by self-assembly in toluene) encapsulating FeCl_3_ within their P4VP core were spread onto cleaned silicon wafers by spin coating [48]. Oxygen plasma treatment was needed to convert the Fe^3+^ precursor into iron oxide and to remove the polymer template. The γ-Fe_2_O_3_ crystals formed at 1.0, 0.5, and 0.2 of FeCl_3_/P4VP molar ratios appeared on the TEM images (Figure 25a) as 25.0, 18.0, and 6.0 nm sized particles, respectively, arranged in a hexagonal self-assembled pattern. The wide range of IONP diameters obtained and the constant spacing of the IONP patterns in all three cases demonstrated that PS-*b*-P4VP is a powerful template that effectively controls the nucleation and the growth of the particles. AFM (Figure 25b) and field emission-scanning electron microscopy (FE-SEM) imaging (Figure 25c) revealed the hemispherical topology of the IONPs. Contrary to what was expected, the annealing process aiming to improve the crystallinity of the particles did not cause irregular shapes but slightly reduced the average diameter and height of the particles for all the samples with different FeCl_3_/P4VP ratios, as shown in the tilt-view image of Figure 25c. The 25.0 nm-sized particles exhibited ferrimagnetism with normalized magnetization *M*_s_ = 100 emu/g and *H*_c_ = 50 Oe, while the 6.0 nm crystals possessed 30 emu/g saturated magnetization and zero hysteresis, corresponding to superparamagnetic properties. The impressive variation in the magnetic properties of the particles obtained was achieved by only by changing the FeCl_3_/P4VP feed ratio in the mixture.

#### 4.5.2. Monolayer Films of Polystyrene-*block*-Poly(Ethylene Oxide) (PS-*b*-PEO) Copolymers 

**Figure 26:**
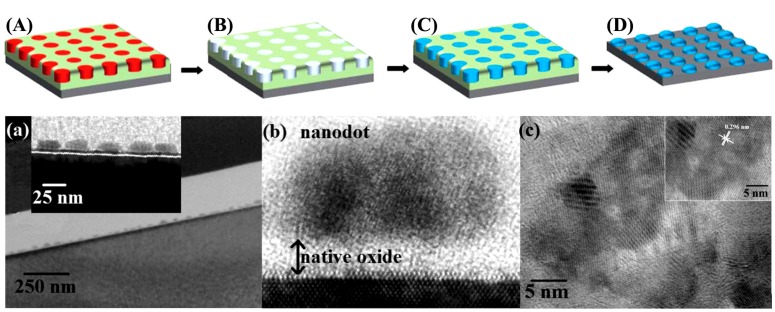
Schematic illustration of the fabrication of oxide nanodots: (**A**) highly ordered PS-*b*-PEO thin film prepared by a solvent annealing process; (**B**) nanoporous template produced by activation of the PEO cylinders upon exposure to ethanol at 40 °C for 15 h; (**C**) the metal oxide precursor diffuses into the cylinders after spin coating the metal nitrate solution; and (**D**) oxide dots remaining after the ultraviolet (UV)/ozone treatment. (**a**) TEM cross-sectional image of iron oxide nanodots; the inset shows a higher magnification image; (**b**) cross-sectional HRTEM image for a single nanodot; and (**c**) HRTEM image for the nanodots after the UV/ozone treatment; the inset shows crystalline fringes corresponding to Fe_3_O_4_. Reprinted with permission from [49]. Copyright 2012 Royal Society of Chemistry.

**Figure 27:**
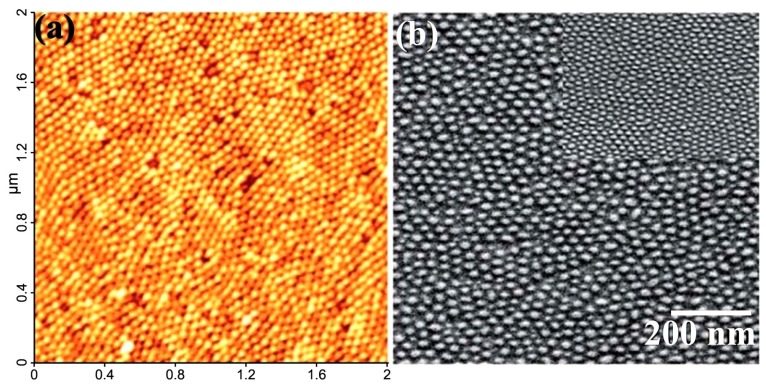
(**a**) AFM; and (**b**) SEM images for hexagonally ordered iron oxide nanodots after UV/ozone treatment. The inset of (**b**) shows the iron oxide nanodots after annealing at 800 °C for 1 h. Reprinted with permission from [49]. Copyright 2012 Royal Society of Chemistry.

Also targeting the preparation of hexagonal arrays of NPs, the *in situ* synthesis of metal oxide NPs through a gas phase oxidation pathway in oriented cylinder structures was investigated by Ghoshal *et al.* [49]. The ordered thin films were prepared using a commercial diblock copolymer of poly styrene (PS) with PEO as the metal-anchoring block. The block weight ratio was selected to yield cylindrical PEO domains 20 nm in diameter and 28 nm in height, regularly distributed within the PS matrix. One of the strategic details differentiating this research from the methods reported earlier was the microphase separation induced by a solvent mixture after spin coating of a toluene solution of the PS-*b*-PEO block copolymer onto cleaned silicon wafers. Annealing with a water/toluene mixture efficiently weakened the preferential interactions between PEO and the substrate, thus allowing the vertical alignment of the PEO cylinders (Figure 26A). A subsequent treatment with an ethanol solution at 40 °C for 15 h (Figure 26B) appeared necessary as an “activation step” for the PEO cylinders to create a functional chemical pattern for nanodot development, which was interpreted by the authors as being due to the crystallization of the PEO blocks induced by ethanol. Then the affinity of PEO for Fe(NO_3_)_3_ as compared to PS allowed selective inclusion of the metallic salt by spin-coating of the 0.4 wt% precursor solution (Figure 26C). Finally, ultraviolet (UV)/ozone treatment (Figure 26D) produced highly ordered Fe_3_O_4_ particles (or CeO_2_ or CuO, depending on the precursor). This treatment not only produced the oxides from the precursors but also removed the polymer template. The Fe_3_O_4_ particles appeared in HRTEM images as nanodots 24 nm in diameter and 9 nm in height, with a regular spacing of 42 nm between their centers. Control over the diameter and the height of the oxide nanodots was attained by varying the precursor concentration, which changed the height and the diameter of the nanodots without affecting their spacing. Precursor solution concentrations exceeding 1% led to saturation of the PEO cylinder template, however. This method nevertheless offers great potential to easily tune the NP size for iron oxide and several other inorganic materials. In addition, the nanodots could be sintered without losing their structural order, as seen on Figure 27. For example, at 800 °C in air, the IONPs shrunk in diameter by 3 nm and in height by 2 nm, as ascribed to oxide densification.

### 4.6. Holey Membranes

#### 4.6.1. PVA-Fe^3+^/Citric Acid (CA)/Ethylene Glycol (EG) Calcination

PVA, containing a high density of hydroxyl functional groups available to complex ferric ions, was employed in a so-called “polymer precursor” (PP) method to produce porous iron oxide films [50]. The first step in the PVA-PP method involved the preparation of a viscous solution of Fe^3+^ ions complexed with PVA and the polyether produced *in situ* by the reaction of CA and EG. The solution was then spin-coated on a Pyrex^®^ glass substrate before repeated calcination to decompose the PVA into gas and yield a uniform porous iron oxide film consisting of sintered 100 nm diameter ellipsoidal particles, with a micro-porosity volume (assessed by the N_2_ adsorption isotherm method) increasing with the PVA/CA weight ratio. A change in crystal structure from α-Fe_2_O_3_ to γ-Fe_2_O_3_ was detected by PXRD and X-ray photoelectron spectroscopy (XPS) measurements when the PVA/CA ratio exceeded 1, which was interpreted as being due to the surface reduction of α-Fe_2_O_3_ into Fe_3_O_4_ crystals. The kinetic constant for this transformation was enhanced by a decrease in Gibbs energy for the reduction due to improved elimination of O_2_ for the higher porosity films.

#### 4.6.2. Loading of the Pores of a Nafion^®^ Membrane Followed by Reduction

In comparison to commonly used methods like coprecipitation or thermal decomposition, fewer studies relied upon a reduction reaction. This route was selected by Yoon *et al.* [51] using a polymeric ion-exchange membrane to produce nanosized magnetic pristine iron particles. The method basically consisted in the diffusion of Fe^3+^ ions into a commercial porous perfluorinated sulfonate ionomer membrane, and the *in situ* reduction with NaBH_4_ into magnetic Fe NPs. This led to the disappearance of the hysteresis loop at 120 K, corresponding to the *T*_B_ for very tiny Fe NPs, as well as ferromagnetism at 10 K and superparamagnetism at 200 K. The observation of 1.5–2.5 nm diameter spherical particles dispersed in a clear polymer background in the TEM images, combined with crystalline lattices revealed by EDS measurements, confirmed the single-domain structure of the iron NPs obtained.

### 4.7. Tridimensional Scaffolds (Macroscopic Samples)

In an encapsulation process, the strength of interactions between the anchoring moieties in a polymer matrix and the NPs is considered the most important criterion preventing the diffusion of the NPs out of the template and leading to successful directed nucleation and growth. One approach suggested realizing this condition was to rely on the large surface area and the complicated inner structure of polymer gel networks to perform the *in situ* synthesis of MNPs. Tridimensional polymeric scaffolds containing further structural hierarchical microstructures offer great potential for size, shape, crystalline structure, and orientation control, and have consequently motivated a large number of studies.

#### 4.7.1. Porous Preformed Carbon Foams (CF)

**Figure 28:**
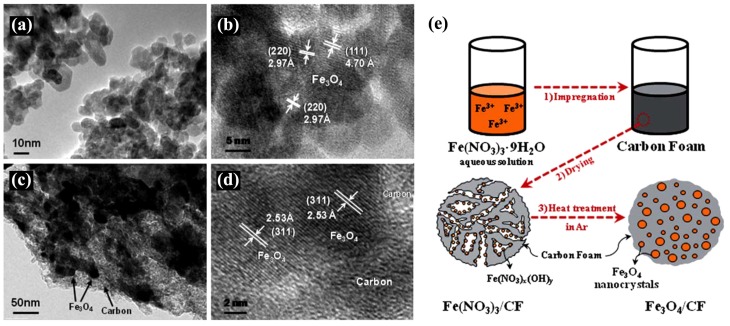
TEM images for unsupported Fe_3_O_4_ NPs synthesized by: (**a**,**b**) the hydrothermal method; (**c**,**d**) the *in situ* formation of Fe_3_O_4_ NPs on the CF composite; and (**e**) schematic representation of the *in situ* formation of Fe_3_O_4_ nanocrystals in the confined pores of carbon foam. Reprinted with permission from [52]. Copyright 2011 Royal Society of Chemistry.

In an innovative study by Yoon *et al.* [52], mesoporous CF produced by the resorcinol-formaldehyde method, with a very large pore volume (0.87 cm^3^∙g^−1^) and specific surface area (834 m^2^∙g^−1^) were loaded with a Fe(NO_3_)_3_ solution by repeated impregnation. After *in situ* thermal decomposition of the precursor at 450 °C under argon, Fe_3_O_4_ nanocrystals were obtained in the confined pores of the carbon template (Figure 28). HRTEM images and XRD patterns confirmed the Fe_3_O_4_ composition of the crystals, which had a non-uniform size and shape distribution in the 5–50 nm range, *i.e.*, larger and broader than the 7–15 mm particles obtained by the conventional (non-templated) polyol synthesis (same precursor but at 180 °C in EG). The width of the XRD peaks also yielded a significantly larger average crystallite size of 21.7 nm (as compared to 10.1 nm for the polyol pathway). Although physical adsorption was invoked rather than chemical interactions, due to the absence of coordinating functional groups, the roles of the carbon foam in orientating the growth of the iron oxide nanocrystals and preventing their agglomeration were clearly shown. When used as anode material for lithium-ion batteries, the as-prepared CF/Fe_3_O_4_ composites exhibited superior charge/discharge capacities as compared to the unsupported materials.

#### 4.7.2. Sponge-Like Polystyrene (PS)/Polyacrylate Copolymer Gel

**Figure 29:**
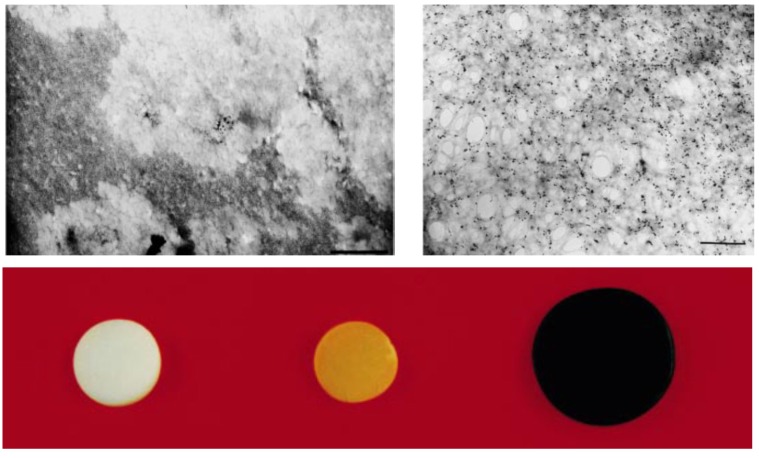
TEM images for sample sections: (**a**) 3.5 wt% Fe-loaded polystyrene (PS)-polyacrylate gel prior to reaction with NaOH (scale bar = 500 nm); (**b**) after reaction with NaOH, showing the distribution and uniformity of the magnetite nanocrystallites (scale bar = 500 nm); and (**c**) synthesis of the magnetic sponge-like copolymer gel. Comparison between: (**c**, **left**) an unloaded polymer gel; (**c**, **middle**) after exposure to a 0.2 M Fe^2+^ solution; and (**c**, **right**) after reaction with NaOH. The gels imaged with a digital camera on (**c**) are in the swollen state. Reprinted with permission from [53]. Copyright 1998 WILEY-VCH Verlag GmbH.

In the early work, the team of Antonietti and Mann [53] successfully developed magnetic iron oxide nanocolloids using elastic sponge-like PS-polyacrylate copolymer gels, providing a mesoporous open-cell structure with carboxylic groups readily coordinating with metallic ions. The hierarchical structures of the micro-spherical pores, whose size could be further adjusted during synthesis, offered enormous surface areas that effectively facilitated the templating and nucleation processes (allowing up to 20 wt% of iron loading). It is worth noting that the gel structure was not changed after the loading step (Figure 29a). There were no crystalline structures in the air-dried composites, but rather amorphous hydrated Fe^3+^ oxide which gradually converted to 24 nm magnetic Fe_3_O_4_ crystals (based on wide-angle X-ray scattering (WAXS) line width analysis). These results were in agreement with TEM analysis of gel sections, showing homogeneous spherical and cubic particles with a mean size of 16.3 ± 9.4 nm trapped in the wall of the gels (Figure 29b). No hysteresis was reported in room temperature measurements, indicating the superparamagnetism of the particles. This pioneering research using an easily tailored material was followed by many other studies on gel templates.

#### 4.7.3. Cross-Linked Poly[Styrene-*co*-(*N*-4-Carboxybutylmaleimide)] Copolymer

Taking advantage of the facile polymerization of styrene and maleimide-based monomers, cross-linked networks were created from poly[styrene-*co*-(*N*-4-carboxybutylmaleimide)] copolymers poly(St-*co*-CBMi) in which CBMi acts as a metal-anchoring moiety. This copolymer was successfully prepared and used for the *in situ* synthesis of IONPs by deposition of Fe^2+^ and multi-step NaOH treatment [54]. The data obtained from XRD analysis after two particle deposition cycles revealed the initial existence of maghemite (γ-Fe_2_O_3_) NPs which were gradually transformed to goethite (α-FeOOH). This was likely not due to oxidation as ascribed by the authors, but rather a result of the unavoidable pH increase in such a multi-step NaOH treatment. The constant value of mean particle size (14 nm) calculated from the Scherrer equation and XRD diffractograms after several deposition cycles indicate that the growth of the particles was inhibited by the constrained architecture of the polymer network. The maghemite content increased after each loading cycle, but the fourth cycle led to agglomeration. The magnetization of the hybrids also increased after each loading cycle, but was maximized after the third one (8.04 emu/g). There was no subsequent change in *M_s_* due to the appearance of the goethite crystals, that are known to be antiferromagnetic. The non-zero *H*_c_ values observed for the hybrids, even at room temperature, were assigned to the same reason, *i.e.* mixing of the two phases giving rise to spontaneous ferrimagnetism.

#### 4.7.4. Semi-Interpenetrating (Semi-IPN) Polymer Networks of Alginate and Poly(*N*-Isopropylacryl Amide) (PNiPAAm)

In an attempt to synthesize IONPs within networks of mixed polymer hydrogels, Hernández *et al.* [55] initially prepared semi-IPN polymer networks from alginate bringing chelating carboxylate groups and PNiPAAm providing thermo-sensitivity to these materials. Cylindrical samples cut from the semi-IPN were submerged into a 1:2 solution of Fe^2+^ and Fe^3+^ ions, and then into a NH_3_ solution for the *in situ* preparation of semi-IPN ferrogels. For samples with different numbers of alkaline bath treatments, the phase type of the IONPs within the gels was determined to be a combination of magnetite and maghemite based on the diffraction patterns in wide angle X-ray analysis and characteristic peaks in Raman spectroscopy. The diameters calculated from the Debye-Scherrer equation for the (311) Bragg peak suggested larger sizes for the NPs seeded in the polymer networks (*D*_311_ = 10.1 ± 2.5 nm and *D*_311_ = 11.2 ± 2.8 nm for one and two alkaline treatments, respectively, as compared to *D*_311_ = 6.7 ± 1.6 nm for NPs created in an alginate solution.

#### 4.7.5. Cross-Linked Polyacrylamide (PAAm) Hydrogels

**Figure 30:**
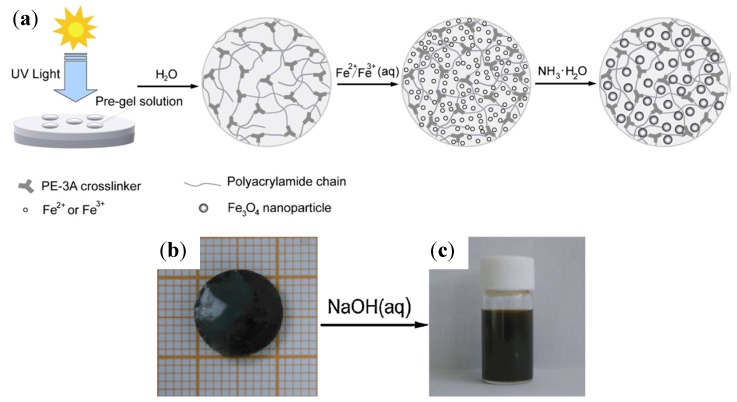
(**a**) Schematic representation; **(b)** appearance of a magnetic hybrid hydrogel obtained by photo-polymerization and *in situ* coprecipitation; and (**c**) in the second step, the ferrogel was exposed to NaOH to hydrolyze the cross-link points and induce the release of water-dispersible IONPs. Adapted with permission from [56]. Copyright 2011 Elsevier B.V.

**Figure 31:**
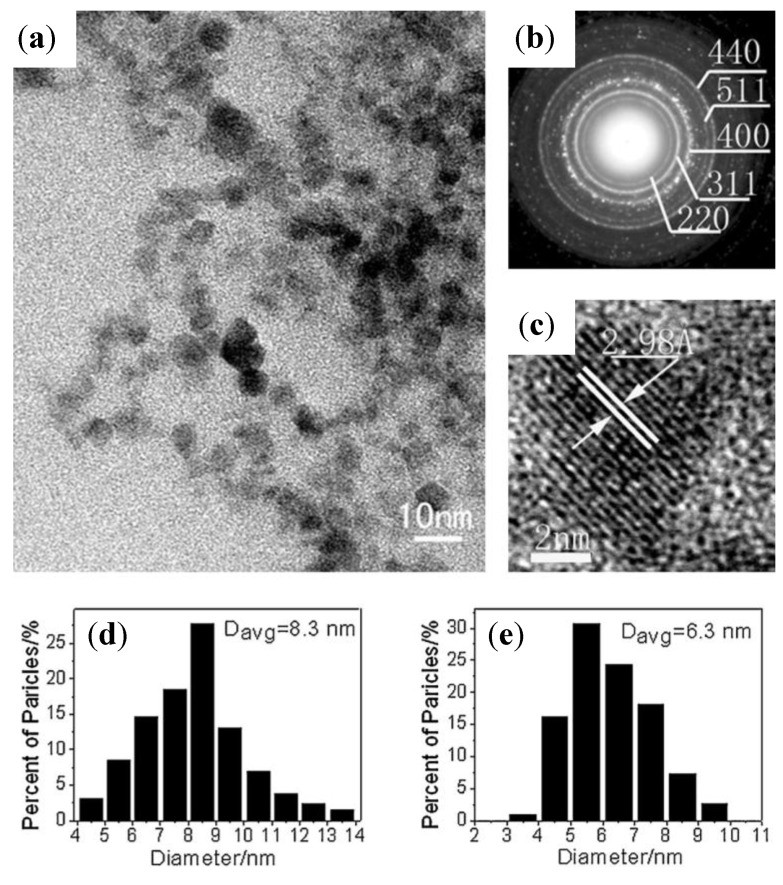
(**a**) TEM image; (**b**) SAED pattern for magnetic Fe_3_O_4_ NPs synthesized by coprecipitation of iron salts within polymer disk; (**c**) typical HRTEM image for a single Fe_3_O_4_ NP, and size distribution of the Fe_3_O_4_ NPs for samples containing the cross-linker at: (**d**) 0.46 mol% and (**e**) 6.14 mol%. Reprinted with permission from [56]. Copyright 2011 Elsevier B.V.

To prepare water-dispersible magnetite NPs, Xiong and Sun applied an *in situ* synthesis approach involving cross-linked PAAm hydrogels and NaOH solution treatments. As schemed on Figure 30a, the PAAm hydrogel was photo-polymerized with pentaerythritol triacrylate (PE-3A) as a hydrolysable cross-linker and BDMBP as photo-initiator [56]. The polymer network was expected to act as template possessing both sufficient capacity for ion impregnation and a restrained mesh structure limiting the growth of MNPs and preventing their aggregation. Magnetite NPs with narrow size distribution were obtained by this method, as seen on Figure 31, with a size inversely related to the cross-linker density (8.3 nm for 0.46 mol% PE-3A, 6.3 nm for 6.14 mol%). In a second step, the hydrolysis of the acrylic ester cross-linker PE-3A in alkaline water liberates well-dispersed hydrophilic MNPs (Figure 30c). Interestingly, XPS results showed the existence of a thin layer of polymer chains bearing carboxylate and acrylamide groups covering the surface of these NPs, which explains their long term stability in water. Hysteresis and *H*_c_ were observed at 5 K, indicating ferromagnetism, but the particles were superparamagnetic at 300 K with negligible *H*_c_. The saturated magnetization was 44.6 emu/g at 300 K.

#### 4.7.6. Hydrogels of Poly(2-Acrylamido-2-Methyl-1-Propansulfonic Acid) (PAMPS) and P4VP

Hydrogels prepared by radical copolymerization, either with a photo-initiator for PAMPS [57], or a redox (persulfate) initiator for poly(4-vinyl pyridine) (P4VP), poly(2-hydroxyethyl methacrylate) p(HEMA), and poly(4-vinyl pyridine-*co*-2-hydroxyethyl metacrylate) (P4VP-*co*-HEMA) [58], were impregnated with FeSO_4_ and FeCl_3_ and employed in a coprecipitation reaction to create magnetic composite hydrogels. These hydrogels, designed for water remediation, were able to absorb toxic heavy metal ions efficiently in aqueous media. Unfortunately, no information was provided on the size and the morphology of the magnetic particles obtained.

#### 4.7.7. PAAm Hydrogels

**Figure 32:**
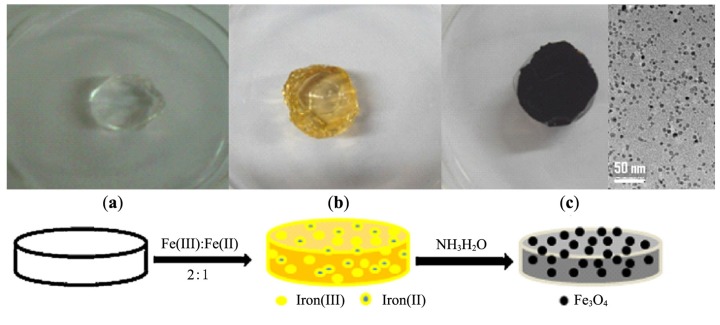
Schematic views and pictures of polyacrylamide (PAAm) hydrogel networks at three steps of loading by magnetite NPs: (**a**) swollen hydrogel; (**b**) iron ion-loaded hydrogel; and (**c**) magnetite NPs in the hydrogel matrix, inset: TEM image showing well-dispersed MNPs of uniform size. Reprinted with permission from [59]. Copyright 2009 Elsevier B.V.

PAAm hydrogels were employed as templates for the coprecipitation of Fe^2+^ and Fe^3+^ to create pH-responsive magnetic hydrogels [59]. The magnetite particles obtained were 3–5 nm in size and homogeneously distributed inside the hydrogel network (Figure 32). The population of Fe_3_O_4_increased for higher iron ion feeds (from 25 wt% to 100 wt% relatively to PAAm), indicating the good loading capacity of the template. Despite a low *M*_s_ value of 4 emu/g ascribed to the low size range of the MNPs and to the large weight proportion of the polymer matrix, the composite exhibited superparamagnetism with only a small *H*_c_ of 14.8 Oe.

### 4.8. Dispersed Colloids (Microscopic)

#### 4.8.1. Poly(*N*-Isopropyl Acrylamide-*co*-Acrylic acid-*co*-2-Hydroxyethyl Acrylate) (Poly(NIPAM-*co*-AA-*co*-HEA)) Microgels Cross-Linked with *N*,*N*′-methylene Bisacrylamide (BIS)

**Figure 33:**
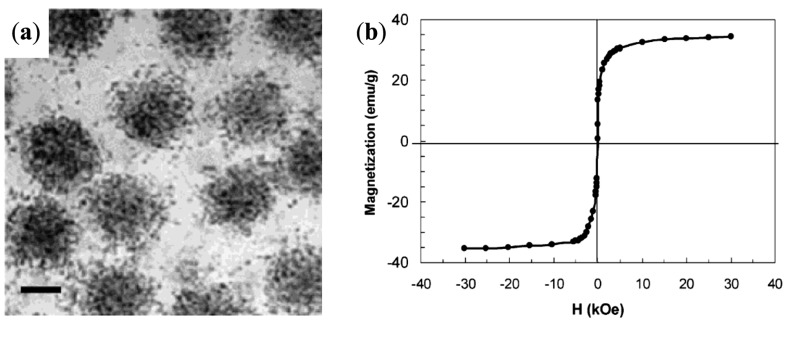
(**a**) TEM image for hybrid poly(*N*-isopropyl acrylamide-*co*-acrylic acid-*co*-2-hydroxyethyl acrylate) (poly(NIPAM-*co*-AA-*co*-HEA)) microgels loaded with 0.618 g Fe_3_O_4_/g polymer; the scale bar is 150 nm; and (**b**) magnetization curve at 300 K. Reprinted with permission from [60]. Copyright 2004 American Chemical Society.

Sub-micrometer gels of poly(NIPAM-*co*-AA-*co*-HEA), prepared by suspension polymerization and cross-linked with BIS, were loaded with ferrous iron ions in an ion exchange process [60]. A post-loading oxidation step with H_2_O_2_ was used to produce the IONPs (Figure 33a). The size of these microgels was successfully controlled by the polymerization conditions: a rise of pH from 2.3 to 9.2 transformed the PAA blocks from a highly compact and weakly hydrophobic conformation to a highly hydrophilic and expanded coil one, and greatly increased the repulsive forces between COO^−^ groups, leading to larger particle sizes (from 200 nm to 600 nm). Not surprisingly, the concentration of COOH functionalities in the polymer composition was the dominant parameter affecting the size and the loading capacity of the microgels. However, the Fe^2+^ precursor concentration and the pH value range useable for the reactions were limited by the solubility product of Fe(OH)_2_ (*K*_s_ = 4.87 × 10^−17^): for instance, the maximum allowable pH value was 7.1 for [Fe^2+^] = 1.5 mM. Interestingly, the size distribution of the NPs was relatively narrow (e.g., 8.5 ± 1.0 nm diameter by TEM). These Fe_3_O_4_ NPs trapped in the 500 nm diameter microgels exhibited superparamagnetic properties at 300 K (both the remnant field and the *H*_c_ were zero) with a specific *M*_s_ of 32.4 emu/g (Figure 33b).

#### 4.8.2. Acetoacetoxyethyl Methacrylate (AAEM)-*N*-Vinylcaprolactam (VCL) Microgels

**Figure 34:**
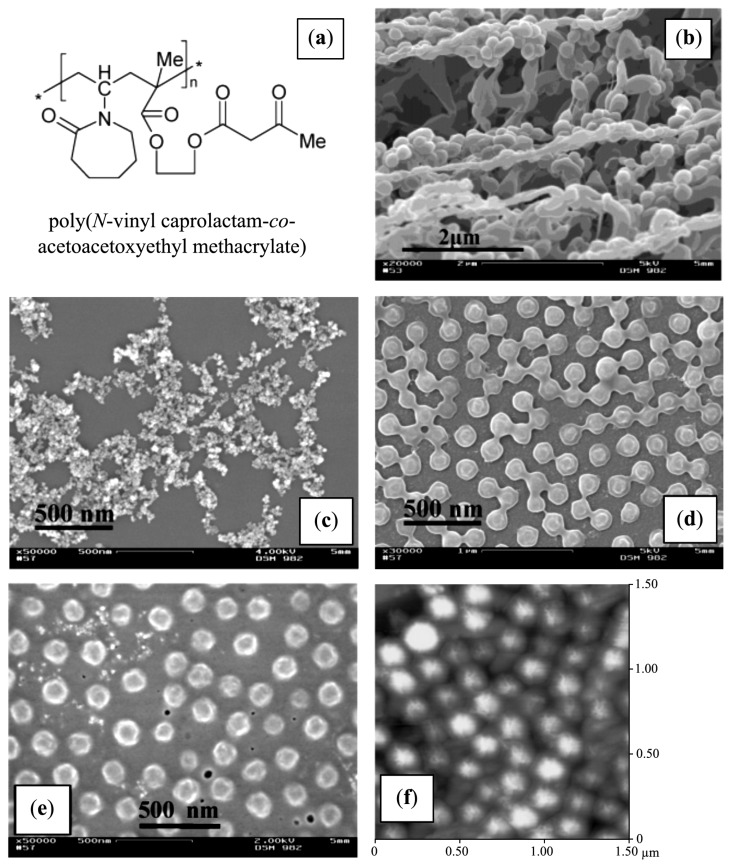
(**a**) Chemical structure of an *N*-vinylcaprolactam (VCL)/acetoacetoxyethyl methacrylate (AAEM) copolymer; and SEM images for: (**b**) empty cross-linked microgels; (**c**) magnetite NPs; (**d**) composite microgels with 4% magnetite; (**e**) SEM; and (**f**) AFM images for microgels with 9.4% magnetite (the height scale of the AFM image in the height mode is 0–100 nm). Reprinted with permission from [61]. Copyright 2004 American Chemical Society.

An interesting characteristic of these systems is that the chemical composition of the microgels can be adjusted to provide additional properties to the MNPs. One example of this is the work of Pich *et al.* [61], who successfully employed microgels obtained by the copolymerization of AAEM and VCL to synthesize thermosensitive magnetic hybrids (Figure 34). It is common knowledge that thermoresponsive polymers such as PVCL can extensively and reversibly swell in appropriate solvents at temperatures below the LCST, but will shrink rapidly above the LCST. The PVCL-rich shell of the microgels, exhibiting an LCST, thus offered the possibility to control their size through temperature changes, while the AAEM-rich portions provided a high iron loading capacity via bidendate coordination. The observed non-monotonic increase of *R*_h_ of the microgels for increasing magnetite loadings was explained in terms of the chemical interactions formed. The slight decrease in *R*_h_ observed when increasing the Fe_3_O_4_ loading from 0 wt% to 7 wt% was attributed to the multivalent complexation between the β-diketone groups of AAEM and the Fe^2+^/Fe^3+^ ions linking simultaneously several AAEM units, thus leading to additional cross-linking and shrinkage of the microgels. When the loading level surpassed 7 wt%, the repulsive forces among crowded Fe_3_O_4_ particles led to swelling of the microgels (increase in hydrodynamic size). An increase in magnetite content (e.g., from 4% to 9.4%), leading to larger magnetite-filled microgel cores, also affected the colloidal stability of the microgels in suspension as measured by their sedimentation velocity. Superparamagnetism was reported, with plateau values of the magnetization curve corresponding to 75 emu/g Fe_3_O_4_. The variation in the magnetic core size *versus* magnetite-to-polymer content in the hybrid microgels was not investigated in this study.

#### 4.8.3. Sulfonated Copolymer Beads

Polymer beads produced by the suspension copolymerization of styrene and a small amount of divinylbenzene were sulfonated to add complexing moieties to the polymer chains and facilitate the encapsulation of Fe^3+^ and Co^2+^ cations for the synthesis of MNPs *in situ* [62]. The presence of a CoFe_2_O_4_ spinel phase was confirmed from its characteristic XRD peaks. TEM images obtained from ground powder displayed 7 nm, nearly spherical particles well-distributed within the polymer matrix. The absence of hysteresis, remanence and *H*_c_ demonstrated the superparamagnetic character of the hybrids, the saturated magnetization being also low (0.818 emu/g) due to a small amount of magnetic content in the hybrid beads.

#### 4.8.4. Poly[Poly(Ethylene Glycol Diacrylate-*co*-Acrylic acid) (PEGDA-*co*-PAA) Copolymer Hydrogels Prepared by Microfluidics

**Figure 35:**
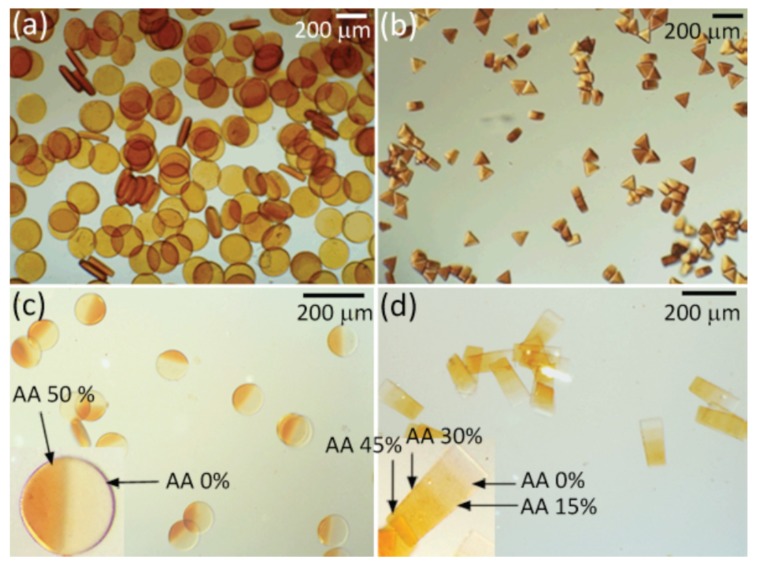
Optical images for various magnetic microparticles: (**a**) homogenous magnetic disks; (**b**) homogenous triangular particles; (**c**) Janus disks; and (**d**) gradient particles. Reprinted with permission from [63]. Copyright 2012 American Chemical Society.

**Figure 36:**
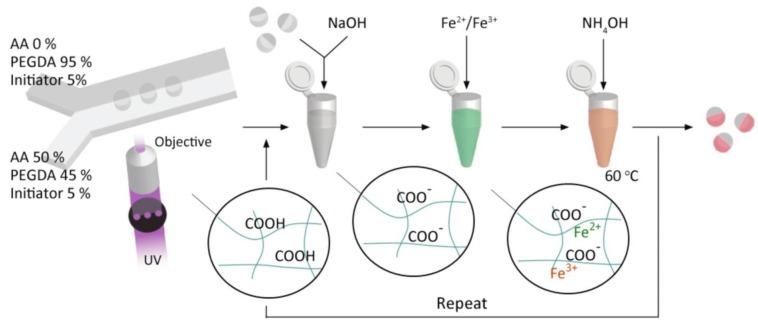
Schematic representation of the coprecipitation process in photo-polymerized microgels with ~20 µm diameter prepared in a microfluidic channel. After deprotonation in 0.5 M NaOH and rinsing with 0.5% Tween 20 to reach pH 7, 0.2 M FeCl_3_ and 1 M FeCl_2_ solutions were mixed with microgels at a Fe^3+^:Fe^2+^ ratio of 1:75. After diffusion of the iron ions in the polymer particles, excess salts were removed. After the addition of NH_4_OH at 60 °C, MNPs were nucleated and grown *in situ* before a final rinse with Tween 20. All these steps were repeated several times for successive growth cycles. Reprinted with permission from [63]. Copyright 2012 American Chemical Society.

Janus particles, with their structure combining two distinct materials on the opposite sides of the NP, were designed in the groups of Doyle and Hatton [63] to work as selective nanoreactors for the *in situ* synthesis of magnetic species. Figure 35 shows Janus-like and other non-spherical hydrogel magnetic particles prepared by stop-flow lithography (SFL), with one part of PEG diacrylate (PEGDA) non-interacting with iron salts, and another part constituted of a copolymer of PEGDA and acrylic acid (AA) to coordinate the iron ions. Since the polymerization was initiated by a photosensitive radical initiator, the shape of the hydrogels could be varied at will (e.g., disks, triangles, *etc.*) by an appropriate mask shaping the UV beam illuminating the reactants in a Y-shaped laminar flow chamber (Figure 36). Then the transfer of the minigels into an alkaline solution converted the COOH groups into COO^−^ and induced swelling by electrostatic repulsion between the anionic carboxylate groups. Interestingly, the Fe^3+^/Fe^2+^ feed ratio used (1:75) was much lower than the required 2:1 stoichiometric ratio due to the different adsorption affinities of these two ions toward COO^−^. The iron content of the particles was increased by repetition of the loading process, which allowed the adsorption of more ions at unoccupied nucleation sites and/or the surface of the magnetic particles, leading to larger, visibly darker and more magnetic particles. The hydrogel particles obtained after eight loading cycles reached an *M*_s_ value of 42 emu/g. It is worth noting that the maximum magnetic domain diameter reported was 5.7 nm (but with a high σ of 0.3–0.4), which was considered to be limited by the swollen mesh size of the polymer network. This suggests partial size control for the MNPs synthesized *in situ* in such hydrogel scaffold.

#### 4.8.5. Multi-Responsive Microgels Made from VCL, AAEM and Vinylimidazole (VIm)

**Figure 37:**
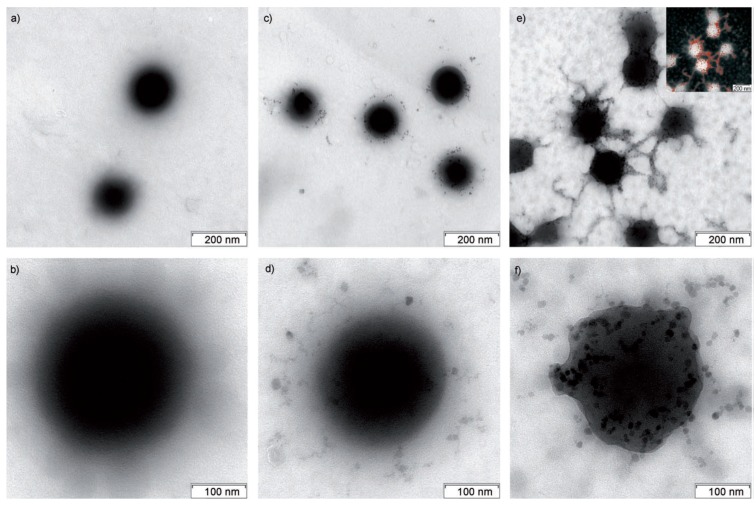
TEM images of microgels for different magnetite contents: (**a**,**b**) no magnetite; (**c**,**d**) 8.4 wt%; (**e**,**f**) 15.4 wt%. Inset: EDX iron-mapping image. Reprinted with permission from [64] Copyright 2007 Wiley-VCH Verlag GmbH & Co., Weinheim.

The wide range of polymeric materials available in terms of number and structural variety, but also in terms of physical and chemical characteristics, have inspired many researchers to look for novel materials with combining different properties. To prepare “multi-stimuli-responsive” magnetic hybrid microgel particles, Bhattacharya *et al.* [64] thus performed coprecipitation reactions of iron ions in the presence of microgels obtained by the batch copolymerization of VCL, AAEM, and VIm, cross-linked with BIS. The VCL-*co*-AAEM-*co*-VIm hybrid microgels were expected to exhibit thermosensitivity brought by the VCL units, pH responsiveness ascribed to the AAEM-rich core, and magnetic switchable properties owning to encapsulated iron oxide particles. Unfortunately, the concentration of VIm units in the copolymer was found to strongly influence the swelling behavior and the transition temperature of the hydrogels. Shrinkage of the hybrid particles was also observed at higher magnetite contents (which led to partial collapse of the network in some cases), in agreement with a previous study [61]. The magnetite particles had a diameter of *ca*. 10 nm, as determined from TEM images (Figure 37). The highest *M*_s_ measured was 30 emu/g for the highest loading obtained of 15.4 wt% magnetite. Evidence for pH responsiveness and temperature-sensitive properties was shown for the hybrids, indicating the success of monomer blending to obtain copolymer microgels with combined characteristics and the creation of multifunctional materials.

### 4.9. Preformed Microspheres in Organic Solvents

#### PS Microsphere Swollen in CHCl_3_

**Figure 38:**
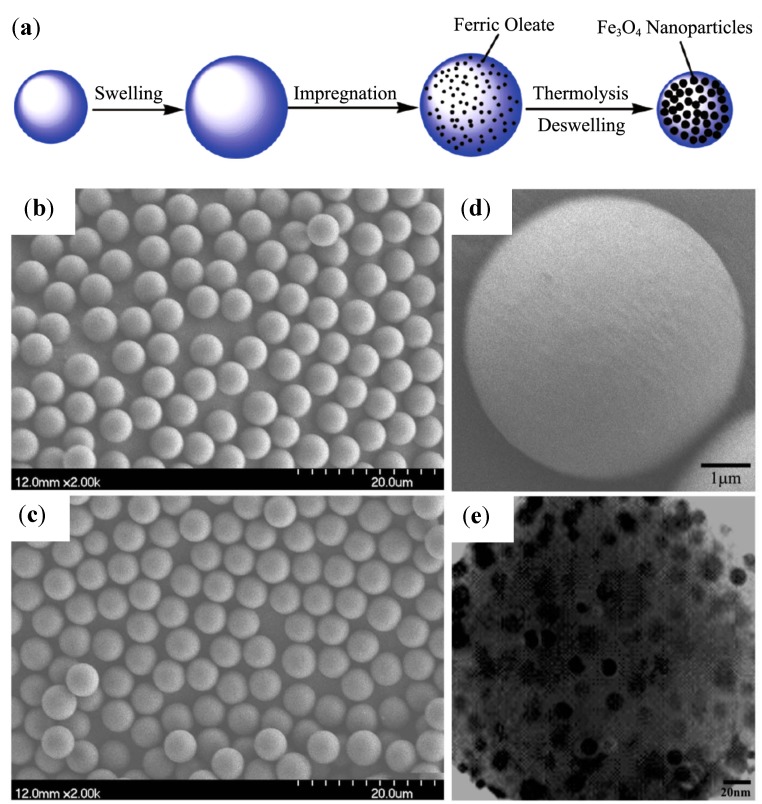
(**a**) Schematic representation of the preparation of Fe_3_O_4_ NPs embedded in PS microspheres by thermal decomposition of Fe(oleate)_3_ at 300 °C; SEM images for: (**b**) the polymer seed microspheres prepared by dispersion polymerization; (**c**) the magnetic polymer microspheres prepared by swelling and thermolysis; (**d**) the outer surface of magnetic polymer microspheres; and (**e**) TEM image for the ultramicrotomed magnetic polymer microspheres, showing the location of the IONPs in the microspheres. Reprinted with permission from [65]. Copyright 2009 American Chemical Society.

Interestingly, even water-insoluble cross-linked PS microspheres were found to be capable of serving as hosts for the thermal decomposition of encapsulated ferric tri-oleate, after swelling of the microspheres in an organic solvent (CHCl_3_) to facilitate the impregnation [65]. The magnetic character of the NPs obtained strongly depended on the decomposition temperature used, since the dissociation of the Fe(oleate)_3_ complex only started around 200–240 °C but was completed at 300 °C, while crystal growth only took place above 310 °C and led to strong magnetism. TGA results showed that decomposition of the polymer matrix occurred between 380 °C and 450 °C, yielding an inorganic content of 15.9 wt% for the composite spheres. SEM and TEM images (Figure 38) revealed nearly spherical IONPs with a mean diameter of 11 nm and low size dispersity (21% standard deviation), distributed uniformly within the network serving as template, by allowing nucleation but inhibiting the overgrowth of NPs. XRD analysis confirmed the Fe_3_O_4_ composition of the iron oxide. The particles exhibited superparamagnetic characteristics at room temperature (*M*_s_ = 12.6 emu/g and *T*_B_ = 171 K), with excellent magnetic responsiveness but readily redispersing after removal of the magnetic field.

## 5. Conclusions

In recent years, multiple routes have been explored for the synthesis of IONPs. This includes the alkaline coprecipitation of ferric and ferrous salts, the oxidation of Fe_2_SO_4_, the thermal decomposition of iron complexes, the polyol route, and hydrothermal processes in various organic templates, as summarized in Table 5. One feature common to all the systems explored so far is the ability of one of the components used to interact with the iron oxide precursor, most often through coordination with the iron ions. A number of the *in situ* syntheses did not give results significantly different from bulk synthesis methods, as these were realized under similar physicochemical conditions (pH, temperature, *etc.*). For instance, syntheses by alkaline coprecipitation leading to IONPs with rock-like shapes and a broad distribution of diameters around 7 nm do not make a strong case for a true templating effect by the organic matrix. In contrast, several investigation listed in this review (in particular those using well-defined polymer structures) highlighted a significant influence of the polymer/precursor ratio on the outcome of the synthesis. This is the case, for instance, for tridimensional networks such as the hydrogels, for which the importance mesh size was recognized as a parameter limiting the diameter of the MNPs grown *in situ*. It was also demonstrated on several occasions that strong ligands (such as phosphate groups) directed the synthesis towards lower particle sizes, which seems to be a general well-known trend in coordination chemistry that can be ascribed to hampering of ion diffusion through the ligand shell during the growth of nanocrystals. On the contrary, other studies reported MNP diameters that increased from 10 nm to 20 nm with the template. This is the case for chemical reactions performed at high temperatures, were an important property required from the organic template is to prevent the aggregation of the growing nanocrystals. This protecting effect was observed in particular in the last study reported, where a PS matrix could withstand temperatures as high as 310 °C for the thermal decomposition of an iron-oleate complex.

Regarding shape control, several studies claimed success of the organic templating method to orient the synthesis towards rod-like NPs. However, caution should be used in that the physicochemical conditions used may themselves favor the precipitation of antiferromagnetic ferrihydrite phases such as goethite or akaganeite. A more convincing case of needle-like cobalt ferrite microparticle formation was reported for a microemulsion process, based on the oriented aggregation of nanocrystals. One interesting aspect that can be brought by polymer-templated synthesis is the ability to prepare NPs with highly magnetic properties (useful for MRI contrast or magnetic hyperthermia) directly covered by a polymer shell, providing colloidal stability and stealth properties in biological media. This was achieved in several investigations by employing either double hydrophilic or amphiphilic copolymers, in particular those forming micelles with a dense core strongly binding to the precursor species and a shell with multiple hydrophilic arms providing steric repulsion.

In all these studies, both with macroscopic matrices and individually dispersed objects, loading of the inorganic precursor into the adsorption sites was critical and needed to be optimized (by iterative process or by careful removal of excess Fe^2+/3+^ ions) to achieve a true templating effect.

**Table 5:** Summary of the different organic templates used as host matrices for the synthesis of MNPs. In this review, the examples gathered from the literature were sorted according to their chemical nature and structural properties (e.g., geometry, dimensionality, size). DEX: dextran; Alg: alginate; PNiPAAm: poly(*N*-isopropylacrylamide); PVA: poly(vinyl alcohol); PVP: polyvinylpyrrolidone; P4VP: poly(4-vinylpyridine); PMGI: poly(methyl glutarimide); DHBC: double-hydrophilic block copolymer; PGA: poly(glycerol acrylate); PAsp: poly(aspartic acid); PAEMA: poly[2-(acetoacetoxy) ethyl] methacrylate; PGMA: poly(glycerol methacrylate); PDMAEMA: poly[(*N*,*N*-dimethylamino)ethyl methacrylate]; and PAMPS: poly(2-acrylamido-2-methyl-1-propansulfonic acid).

**Surfactants**: –COO^−^, –SO_3_^−^, –SO_4_^−^ **Natural polysaccharides**: DEX, DEX sulfate, Alg, DEX-*b*-PMAA-*b*-PNiPAAm	**Synthetic linear polymers**: PVA, PVP, P4VP, PEO, PAA, PMAA, PMGI **Linear DHBC or amphiphilic copolymers**	**Hydrophilic blocks**: iron-complexing (PGMA, PGA, P(norbornene-methanol or di-carboxy), PMAA, PAA, PAsp, PAEA, PAEMA) or repulsive block (PEO, PEOGMA, PDMAEMA)	**Hydrophobic blocks**: PMMA, PNOR, PVBP **Tri-blocks**: Pluronics^®^, PI-*b*-PCEMA-*b*-PtBA
**Three-dimensional scaffold macroscopic matrices**: Carbon foam, sponge-like PS-*co*-PAA, P(S-*co*-CBMi) networks, Alg/PNiPAAm semi-IPN, PAAm, PAMPS, P(4VP-*co*-HEMA) hydrogels	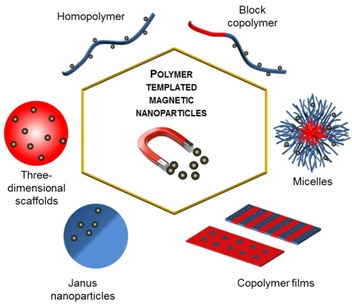		**Star-like** (PPO-*b*-PAA, PEO-*b*-PS) **Comb-like** (PPEGMEA-*g*-PMAA, PEO-*g*-PAA) **Spherical micelles** (PS-*b*-P2VP) **Cylindrical multi-molecular/unimolecular micelles**
**Dispersed colloids**: microgels, hydrophobic PS microbeads	**Janus, gradient or triangle**: PEGDA, PVCL-*co*-AAEM core-VIm shell microgels	**Lamellar** or **hexagonally ordered cylinders** (PS-*b*-P2VP, PS-*b*-PEO) **Membranes** (PVA, Nafion^®^)	**Microemulsions**: hexane/Triton^®^-X/Chitosan, CTAB

## Abbreviations and Acronyms

acac: acetylacetonate
AAEM: acetoacetoxyethyl methacrylate
ADR: antitumor drug adriamycin
AFM: atomic force microscopy
Alg: alginate
AuNR: gold nanorods
BIS: *N*,*N*′-methylene bisacrylamide
CA: citric acid
CBMi: *N-*4-carboxybutylmaleimide
CD: cyclodextrin
CF: carbon foams
CNT: carbon nanotube
CHP: continuous hydrothermal processing
CTAB: cetyl trimethylammonium bromide
DEX: dextran
DHBC: double-hydrophilic block copolymer
DLS: dynamic light scattering
EDS: energy-dispersive X-ray spectroscopy
EFTEM: energy filtering transmission electron microscopy
EG: ethylene glycol
FC: field cooled
FE-SEM: field emission-scanning electron microscopy
FTIR: Fourier transform infrared spectroscopy
G: 1,4-linked α-l-guluronic acid
GDA: glutaraldehyde
Gly: glycidyl
*H_c_*: coercivity
HEA: 2-hydroxyethyl acrylate
HGMS: high-gradient magnetic separator
HRTEM: high resolution transmission electron microscopy
IONP: iron oxide nanoparticle
LCST: lower critical solution temperature
M: 1,4-linked β-d-mannuronic acid
MA: maleic acid
MNP: magnetic nanoparticle
mPEG: poly(ethylene glycol) monomethyl
MRI: magnetic resonance imaging
*M_s_*: saturation magnetization
NIR: near infrared radiation
MW: molecular weight
NaDBS: sodium dodecylbenzenesulfonate
NP: nanoparticle
OE: octyl ether
P2VP: poly(2-vinylpyridine)
P4VP: poly(4-vinylpyridine)
PAA: poly(acrylic acid)
PAEA: phosphonic acid ethyl acrylate
PAEMA: poly[2-(acetoacetoxy) ethyl] methacrylate
PAAm: polyacrylamide
PAMPS: poly(2-acrylamido-2-methyl-1-propansulfonic acid)
PAsp: poly(aspartic acid)
PBO: poly(ethylene-*co*-butylene)
PCEMA: poly(2-cinnamoylethyl methacrylate)
PDMAEMA: poly[(*N*,*N*-dimethylamino)ethyl methacrylate]
PEG: poly(ethylene glycol)
PEGDA: poly(ethylene glycol) diacrylate
PEGMA: poly (ethylene glycol methyl ether methacrylate)
PEO: poly(ethylene oxide)
PGA: poly(glycerol acrylate)
PGMA: poly(glycerol methacrylate)
PI: polyisoprene
PION: pegylated iron oxide nanoparticle
PMAA: poly(methacrylic acid)
PMAA-PTTM: trithiol-terminated poly(methacrylic acid)
PMGI: poly(methyl glutarimide)
PnBA: poly(*n*-butyl acrylate)
PNiPAAm: poly(*N*-isopropylacrylamide)
PNOR: poly(norbornene)
PNORCOOH: poly(norbornene dicarboxylic acid)
PNORMEOH: poly(norbornene methanol)
POEGA: poly(oligoethylene glycol acrylate)
POEGMA: poly(oligo(ethylene glycol) methacrylate
PP: polymer precursor
PPEGMEA: poly(ethylene glycol) methylether acrylate
PPO: poly(propylene oxide)
PS: poly styrene
PSS: poly (styrene sulfonate)
PtBA: poly(*tert*-butyl acrylate)
PVA: poly(vinyl alcohol)
PVBP: poly(4-vinylbenzylphosphonate)
PVP: polyvinylpyrrolidone
PXRD: powder X-ray diffraction
RAFT/MADIX: xanthate reversible addition-fragmentation chain-transfer
*R*_h_: hydrodynamic radius
ROMP: ring-opening metathesis polymerization
SAED: selected area electron diffraction
SANS: small-angle neutron scattering
SDS: sodium dodecyl sulfate
SEM: scanning electron microscope
semi-IPN: semi-interpenetrating
SFL: stop-flow lithography
SPIONs: superparamagnetic iron oxide nanoparticles
SQUID: superconducting quantum interference device
STEM: scanning transmission electron microscopy
*T*_B_: blocking temperature
TEM: transmission electron microscopy
TGA: thermogravimetric analysis
THBC: triple-hydrophilic block copolymer
TMSMA: (trimethoxysilyl)propyl methacrylate
triEG: triethylene glycol
USPIO: ultra-small superparamagnetic iron oxide
UV-Vis: ultraviolet-visible spectroscopy
VCL: *N*-vinylcaprolactam
VIm: vinylimidazole
VSM: vibrating sample magnetometer
XPS: X-ray photoelectron spectroscopy
XRD: X-ray diffraction
ZFC: zero field cooled
WAXS: wide-angle X-ray scattering
